# LINC01133 can induce acquired ferroptosis resistance by enhancing the FSP1 mRNA stability through forming the LINC01133-FUS-FSP1 complex

**DOI:** 10.1038/s41419-023-06311-z

**Published:** 2023-11-25

**Authors:** Shaowen Wang, Jionghuang Chen, Pengping Li, Yangchao Chen

**Affiliations:** 1 School of Biomedical Sciences, Faculty of Medicine, The Chinese University of Hong Kong, Shatin, NT Hong Kong; 2 Neuromedicine Center, The University of Hong Kong-Shenzhen Hospital, Shenzhen, Guangdong 518053 China; 3 Department of General Surgery, Sir Run Run Shaw Hospital, Zhejiang University School of Medicine, Hangzhou, 310000 China; 4 Department of Thyroid & Breast Surgery, The First People’s Hospital of Xiaoshan District, Xiaoshan Affiliated Hospital of Wenzhou Medical University, Hangzhou, Zhejiang China; 5 Shenzhen Research Institute, The Chinese University of Hong Kong, Shenzhen, 518087 China

**Keywords:** Cell death, Cancer therapeutic resistance, Non-coding RNAs

## Abstract

Due to a lack of research on the critical non-coding RNAs in regulating ferroptosis, our study aimed to uncover the crucial ones involved in the process. We found that LINC01133 could make pancreatic cancer cells more resistant to ferroptosis. A higher expression of LINC01133 was associated with a higher IC50 of sorafenib in clinical samples. Furthermore, we discovered that LINC01133 induced this process through enhancing the mRNA stability of FSP1. CEBPB was the transcription factor to increase the expression of LINC01133. A higher CEBPB could also indicate a higher IC50 of sorafenib in patients with cancer. Moreover, we confirmed that LINC01133 could form a triple complex with FUS and FSP1 to increase the mRNA stability of FSP1.

## Introduction

As a refractory disease with a poor 5-year prognosis, the diagnosis of pancreatic cancer increases by 0.5–1% per year, and pancreatic cancer is predicted to rank second among all cancer-caused deaths in 2030 [1, 2]. Due to a lack of suitable screening methods, most patients diagnosed with the disease are often at a very advanced stage and do not have the opportunity to undergo surgery [1]. Although some combinational cytotoxic therapies show relatively modest benefits, as well as severe side effects, such as fluorouracil, leucovorin, oxaliplatin, and irinotecan (FOLFIRINOX), nab-paclitaxel plus gemcitabine, more adjunctive therapeutic alternatives with less severe side effects, are still urgently needed [3].

The cell death processes can be divided into two forms: programmed cell death (PCD) and non-programmed cell death (NPCD). Among PCD, ferroptosis is a novel modality of PCD, which was proposed by Prof. Stockwell in 2012 [4]. After that, more publications focus on this phenomenon, and the underlying molecular mechanisms are gradually uncovered. They mainly include polyunsaturated fatty acid (PUFA) in the phospholipids (PLs), iron metabolism, antioxidant system, etc. [5–7]. Many diseases, such as cancer, hemochromatosis, cardiomyopathy, liver fibrosis, ischemia-reperfusion-injury, etc., have been confirmed to correlate with ferroptosis [8–11]. For some diseases, ferroptosis seems to contribute to the development, such as ischemia-reperfusion-injury and liver fibrosis. While in cancer, ferroptosis has been reported to be a new weapon for the immune system to inhibit the initiation and development of cancer [10]. It was suggested that ferroptosis could be taken advantage of to combat traditional chemotherapy-insensitive cancer [12]. Despite the fact that ferroptosis has shown very promising anti-tumor efficacy in modified KPC mice [13], the acquired resistance to ferroptosis can still be expected.

LncRNAs belong to the long non-coding RNA family of more than 200 nucleotides. Although most of them cannot code protein, some of them still have been proven to be able to be translated into some functional peptides [14]. According to the HUMAN GENCODE, more than 16,000 lncRNAs are recorded [15]. The way they participate in regulating physiological function is various, including chromatin regulation, transcription regulation, scaffolds, post-transcriptional modification, etc. [14, 16, 17]. However, there are very few studies focusing on the lncRNAs’ regulation of ferroptosis and even fewer on its role in the acquired resistance to ferroptosis.

In this study, we explored the non-coding RNAs in ferroptosis resistance. We validated that LINC01133 could contribute to the acquired resistance to ferroptosis. CEBPB was confirmed to be the transcription factor responsible for the upregulation of LINC01133. We further found out that it was the LINC01133-FUS-FSP1 complex that enhanced the mRNA stability of FSP1 to lead to the acquired ferroptosis resistance.

## Materials and methods

### Cell culture

PAAD cell lines, PANC-1, SW1990, Capan-2, CFPAC-1, and Panc 04.03 were purchased from the American Type Culture Collection (ATCC). PANC-1 and SW1990 cells were grown in Dulbecco’s modified Eagle’s medium (DMEM) supplemented with 50 units/mL penicillin-streptomycin (HyClone, USA) and 10% fetal bovine serum (FBS) (Gibco, USA). CFPAC-1 was cultured in Iscove′s Modified Dulbecco′s medium (IMDM) with 50 units/mL penicillin-streptomycin (HyClone, USA) and 10% FBS (Gibco, USA). Capan-2 was cultured in McCoy’s 5a Medium Modified added with 50 units/mL penicillin-streptomycin (HyClone, USA) and 10% FBS (Gibco, USA). Cells were grown at 37 °C in 5% CO_2_ in a humidified incubator. When the cells had attained 70–80% confluency, the culture media was withdrawn, and the cells were washed once with PBS before being trypsinized with 0.05% trypsin (Gibco). By adding an equivalent volume of culturing media, trypsinization was stopped. The cells were then centrifuged at room temperature for 3 min at 1000 rpm. The pelleted cells were resuspended in culture media and transferred to a fresh cell culture dish. The 293F cell line was purchased from Thermo Fisher and cultured on a shaker at 125 rpm/min at 37 °C in 8% CO_2_ in a humidified incubator.

### RNA isolation

TRIZOL Reagent was used to isolate RNA from the whole-cell lysate. Cells were homogenized in 500 μL TRIZOL Reagent after being rinsed twice with chilled PBS. 100 μL chloroform was added to the sample after 5 min of incubation at room temperature, followed by 15 s of vortexing and 3 min at room temperature. The mixture was then centrifuged for 15 min at 4 °C using a 12,000 g centrifuge. 250 mL isopropanol was added to the aqueous layer in a fresh tube. The mixture was vortexed for 15 s before being incubated for 15 min at room temperature. The mixture was then centrifuged for 12 min at 12,000 RPM. The final RNA was measured with the NanoDrop Spectrophotometer (ND-1000, Thermo Scientific, Rockford, IL) to test its quality and obtain its amount.

### QRT-PCR

QRT-PCR was used to determine gene expression levels. Applied Biosystems’ High-Capacity cDNA Reverse Transcription Kit (500 ng RNA) was used to reverse-transcribe the total RNA. The ABI 7900HT Real-Time PCR machine was used to run qRT-PCR using SYBR Green PCR Master Mix (Applied Biosystems). The qRT-PCR primes include:

LINC01133, 5’-TGGTCCTGCTGTGGTGGAGA-3’ (forward), 5’-GGCTCTGGATTCTTGATACTGTCTTCT-3’ (reverse), CEBPB, 5’-CTTCAGCCCGTACCTGGAG-3’ (forward),

5’-GGAGAGGAAGTCGTGGTGC-3’ (reverse),

ACTB, 5’-AATCGTGCGTGACATTAAGGAGAAG-3’ (forward), 5’-CAGGAAGGAAGGCTGGAAGAGTG-3’ (reverse),

LINC01133 promoter, 5’-TTCCACCTCAGCCTCCCAAGTA-3’ (forward), 5’-GCAGTGACTCATGCCTGTAATCC-3’ (reverse),

GPX4, 5’-TGAAGATCCAACCCAAGGGC-3’ (forward),

5’-AGCCGTTCTTGTCGATGAGG-3’ (reverse),

DHODH, 5’-CCACGGGAGATGAGCGTTTC-3’ (forward), 5’-CAGGGAGGTGAAGCGAACA-3’ (reverse),

FUS, 5’-CTTCGTTGCTTGCTTGCCTGTG-3’ (forward), 5’-CACTGTAACTCTGCTGTCCGTAGG-3’(reverse),

FSP1, 5’-AGTAGTGGGGATAGACCTGAAGA-3’ (forward), 5’-CCACCACGATGAACCGTGA-3’ (reverse),

LINC02806, 5’-CCTCCAAGCAGAAGGAATCCAATACC-3’ (forward), 5’-TGTGGCTGGCGAAGGTGAGT-3’ (reverse),

RND1, 5’-CCTACTACGATAATGTCCGTCCACTCT-3’ (forward), 5’-AGGTCTGTTCGCAGGTCTGTCTT-3’ (reverse),

ULBP1, 5’-GCCAGGATGTCTTGTGAGCATGAA-3’ (forward), 5’-ATGAAGCAGAGGAAGATGATGAGAAGG-3’ (reverse),

BGIG9606_51662, 5’-CGCTGGCTGTCTTAGAAGGATGAA-3’ (forward) 5’-CGGATGTGAAGTGCTGGTGAGA-3’ (reverse),

LOC105378936, 5’-ATTAGTGCTTGGTGCTGTTGTGGAA-3’ (forward), 5’-GGTTCAGGCTGTTGATGAGAATGGT-3’ (reverse),

CircRNA (chr4:25848916|25849486), 5’-AGTGTGCCTTGAGTGGAACATGG-3’ (forward), 5’-TCGTCAGGGAAGTCTGCCTACC-3’ (reverse).

### Plasmid and oligonucleotide transfection

The lipo3000 manufacturer’s procedure for plasmid and siRNA transfection was strictly followed. In a nutshell, 100,000 cells were seeded on a well in the 6-well plates and cultivated overnight at 37 °C. On the next day, 3.75 μL lipofectamine 3000 was added to 125 μL Opti-MEM for a well of the 6-well plate. 10 μL 20 mM siRNA (GenePharma, China) was added to 125 μL Opti-MEM for a well of the 6-well plate. After mixing the diluted lipofectamine with the diluted siRNA, the mixture was incubated for 15 min at room temperature. Next, 250 μL was added to the wells as a transfection mixture. As a result, 10 nM siRNA was used to transfect cells. The transfection mixture was replaced with the complete culture media 6–8 h after transfection. For the plasmid transfection, P3000 was also added to the mixture with other consistent steps. All the siRNAs were ordered from the GenePharma company. SiRNAs used in this study were listed as the following: Negative control (NC) siRNA, UUCUCCGAACGUGUCACGUTT, LINC01133 siRNA1, CUUGAAGUUACCUGAGAAUTT, LINC01133 siRNA3, GUGCCUCUGAGUUUGUUUATT, CEBPB siRNA1, UAAAUAUAGAUAUAUAUACTT, CEBPB siRNA2, UUCUUUAAGCGAUUACUCCTT, FUS siRNA1, UUAUAUUAGCCUGAUUUGCTT, FUS siRNA2, UAAAUAAAGUCAUAGUUUCTT,

### Western blotting

According to the abundance of the specific protein, the total protein ranging from 10 to 50 μg according to its abundance was mixed with 20% (v/v) loading buffer and 5% (v/v) β-mercaptoethanol for 10 min denaturation at 99 °C. Protein samples were loaded onto an SDS-PAGE gel for electrophoresis at the constant voltage of 80 V for 30 min and 100 V for 60 min. Electrophoresis would be stopped when the target protein reached the middle of the gel. Proteins were transferred to the methanol-activated 0.22 μm PVDF membranes (Millipore) using the wet transfer method for 100 min at a constant voltage of 100 V. Following the transfer, the membrane was blocked in 5% non-fatty milk for 60 min at room temperature. Then, the membrane was incubated with the primary antibody of the recommended concentration overnight at 4 °C. Following that, the membrane was washed three times in TBST for 10 min each time and then incubated in HRP-conjugated secondary antibody targeting rabbit IgG, mouse IgG, or goat IgG, depending on the primary antibody. The membrane was treated for 1 min with a horseradish peroxidase (HRP) substrate (cell signaling), and ECL signals were acquired using the Bio-Rad ChemDoc Imaging System. The primary antibodies were as follows:

Beta tubulin (BIOSS, bsm-33034M, 1:10000), DHODH (CST, #80981, 1:1000), GPX4 (Abcam, ab125066, 1:1000), FUS (CST, #67840, 1:1000), FSP1 (Abcam, ab155326, 1:1000), U2AF2 (NOVUS, NBP1-57251, 1:1000), PTBP1 (Abcam, ab133734, 1:1000), CEBPB (Abcam, ab32358, 1:1000).

### Protein extraction

After rinsing the cells twice with ice-cold PBS and removing the PBS, cells were treated with RIPA lysis buffer (containing a Roche protease inhibitor cocktail tablet and a Thermo Fisher phosphatase inhibitor tablet) and incubated on ice for 5 min. A cell scraper was used to collect the cell lysates. And we transferred them to 1.5 mL tubes. After about 20 min of Centrifugation at 16,000 g, the insoluble components were discarded. The supernatant was collected and kept at –80 °C in the refrigerator. The BCA protein assay kit (Thermo Fisher) was used to quantify the protein concentration according to the instructions.

### Chromatin immunoprecipitation assay

The CEBPB binding to the LINC01133 promoter was determined using a ChIP experiment. The cells were collected and processed for cross-linked chromatin in PANC-1, PANC-1 FR, and CFPAC-1 cell lines. The chromatin was broken down into approximately 300 bp pieces using a Bioruptor UCD-200 ultrasound machine from Diagenode. Anti-CEBPB (Abcam, ab32358) and IgG were used to incubate the DNA fragments at 4° overnight. The anti-RNA Polymerase II- GAPDH binding was used as the positive control. The manufacturer’s instructions were followed for RNA elution and purification using the High-Sensitivity ChIP Kit (Abcam, ab185913). The degree of enrichment of the LINC01133 promoter sequence was determined using qRT-PCR.

### RNA stability assay

A total of 20,000 PANC-1, SW1990, PANC-1 FR cells after LINC01133 knockdown, LINC01133 overexpression, or FUS knockdown were seeded to a 24-well plate. After 24 h, cells were treated with either 2 μg/mL actinomycin D (Sigma) or DMSO as a control for 0, 2, 4, 6, and 8 h. RNA was then extracted for qRT-PCR.

### RNA immunoprecipitation

Magna RIPTM RNA-Binding Protein Immunoprecipitation Kit (Millipore) was used to accomplish RNA immunoprecipitation according to the manufacturer’s instructions. Cells were rinsed twice in ice-cold PBS before being lyzed with an equivalent amount of RIP lysis buffer. After washing the magnetic beads twice with RIP wash buffer, they were incubated for 30 min at room temperature with 2 μg antibodies against FUS (CST, #67840). The cell lysate was incubated with a magnetic bead-antibody combination overnight at 4 °C for immunoprecipitation. The beads were then washed six times with RIP wash buffer before being digested with proteinase K at 37 °C for 30 min. Purification of RNA using phenol:chloroform:isoamyl alcohol (15593-031, Invitrogen). The technique qRT-PCR was taken advantage of to detect the LINC01133 and FSP1 mRNA.

### In vitro transcription

MEGA script T7 Transcription Kit was used to perform in vitro transcription of LINC01133 and FSP1 mRNA (Thermo Fisher Scientific). One microgram template DNA was combined with 0.15 μL of biotin-UTP solution purchased from Epicentre, 1 μL of enzyme combination, 1 μL of ATP solution, and 1 μL of CTP solution, 1 μL of GTP solution, 0.9 μL of UTP solution, and 10 μL of RNase-free water. After overnight in vitro transcription at 37 °C, template DNA was digested with DNase at 37 °C for 15 min. Following the manufacturer’s procedure, digestion was stopped using ammonium acetate stop solution, and in vitro transcripted RNAs were purified with phenol:chloroform:isoamyl alcohol solution.

### RNA pull-down assay

Pierce Magnetic RNA-Protein Pull-Down Kit was used to perform an RNA pull-down test to investigate RNA-protein interaction (Thermo Fisher Scientific). 5 μg of in vitro transcripted RNA was heated in 1X RNA Capture Buffer for 2 min at 90 °C, followed by 2 min on ice and 30 min at room temperature. The biotin-labeled RNA was then combined with 75 μL of streptavidin magnetic beads that had been pre-washed twice in Tris-Buffer. The RNA-labeled beads were rinsed twice with Tris-buffer after 30 min of incubation at room temperature. In 1X Protein-RNA Binding Buffer, 200 μg PANC-1 protein was added to the RNA-labeled beads. Overnight, the mixture was incubated at 4 °C with rotation. The RNA-labeled protein beads were then washed twice with Wash Buffer. After incubation at 37 °C for 30 min, eluted RNA-interacting proteins using 50 μL Elution Buffer followed by Western blot.

### Dual-luciferase reporter assay

JASPAR 2022 (https://jaspar.genereg.net/) and PROMO (https://alggen.lsi.upc.es/cgi-bin/promo_v3/promo/promoinit.cgi?dirDB=TF_8.3) were used to identify putative transcription factors. By referring to the binding locations between CEBPB and the LINC01133 promoter region, we first generated the wild-type (WT) and mutant (MUT) LINC01133 promoter plasmids (pGL3- LINC01133-promoter-WT or pGL3-LINC01133-promoter-MUT). Using Lipofectamine 3000, the CEBPB-overexpressed PANC-1 cells were co-transfected with the appropriate plasmids and the Renilla luciferase plasmid. The Dual-luciferase assay kit (Promega) was used to assess luciferase (Firefly/Renilla) activity 48 h after transfection.

### RNA fluorescence in situ hybridization (FISH) and immunofluorescence (IF)

FISH was conducted first before IF staining. Cells were fixed on 15 mm confocal dishes in 4% formaldehyde followed by being permeabilized with 0.1% Triton X-100. Then samples were dehydrated in ethanol (50, 80, and 95% (v/v) series, exposure for 3 min to 500 µL ethanol at each concentration) prior to hybridization. The probes (5’ FAM FSP1:caacgggatggcctgagaac, 5’ Cy3 LINC01133: cttggttaccaccactgatg) purchased from Genepharma company were diluted with the hybridization buffer (5 μL/500 μL). They were denatured in 73 °C for 5 min. Samples were hybridized for 12–16 h at 37 °C using a moisture-sealed slide incubation chamber. Samples were then briefly rinsed with probe-free hybridization buffer preheated to 55 °C. Incubated cells with 1% BSA, 22.52 mg/mL glycine in PBST (PBS + 0.1% Tween 20) for 30 min to block unspecific binding of the antibodies. Incubated cells in the diluted FUS antibody in 1% BSA in PBST in a humidified chamber for 1 h at room temperature. Decanted the solution and washed the cells three times in PBS, 5 min each wash. Incubate cells with the secondary antibody (Thermo Fisher, A27040) in 1% BSA for 1 h at room temperature in the dark. Decanted the secondary antibody solution and washed three times with PBS for 5 min each in the dark. Incubated cells on 1 μg/mL DAPI for 20 min. Rinsed the samples with PBS 5 min each in the dark. Images were acquired using an Olympus IX83-ZDC Inverted Microscope.

### Protein expression and purification

In all, 5–6 × 10^5^ cells/mL HEK293F cells were diluted with medium (1:1) and cultured for 24 h before transfection. 5 mL Opti-MEM and 100 μg FUS-containing pcDNA3.4 (purchased from Guangzhou IGE Biotechnology Ltd) were mixed in a new clean tube (A). Mix 5 mL Opti-MEM and 500 μg TA-293 (500 μL) in a new sterile tube (B). Add B to A and incubate the mix at room temperature for 15 min. Add the complex to the 293F cell. Shake gently to mix well and return the cell to the incubator. Twenty-four hours later, add 0.6 mL KE-293 and 2 mL KT-Feed. Six days later, harvest the cell medium by centrifuge (12,500 rpm, 30 min, 4 °C) and purify the target protein by Ni-NTA. The SDS-PAGE and western blotting were used to validate the purification of FUS.

### TCGA and GDSC data processing

The TCGA dataset (https://portal.gdc.com) was used to retrieve RNA-sequencing expression profiles and clinical data. Based on the most prominent publicly accessible pharmacogenomics database, the Genomics of Drug Sensitivity in Cancer (GDSC) (https://www.cancerrxgene.org/), we predicted the chemotherapeutic response for each sample. The “pRRophetic” R package was used to build the prediction procedure. The half-maximal inhibitory concentration (IC50) of the samples was calculated using ridge regression. The default settings were used for all parameters. The replicate gene expression was averaged as a mean value using the batch impact of combat. R foundation for statistical computing (2020) version 4.0.3 was used to develop all of the analysis.

### Ferroptosis drugs panel

PANC-1, SW1990, Capan-2, CFPAC-1, and Panc 04.03 were treated with different ferroptosis panels. The first ferroptosis panel included erastin (MCE, HY-15763) combined with or without ferrostatin-1 (fer-1) (MCE, HY-100579), deferoxamine (DFO) (MCE, HY-D0903), Z-VAD-FMK (Z- VAD) (MCE, HY-16658B), 3-methyladenine (3-MA) (MCE, HY-19312) and necrostatin-1s (nec-1s) (MCE, HY-14622A). The second ferroptosis panel was cystine depletion with or without fer-1, DFO, Z-VAD, 3-MA, and nec-1s. The cystine-depleted DMEM was made through adding L-Glutamine (Thermo 25030149) and methionine (Beyotime ST1465) to DMEM (high glucose, no glutamine, no methionine, no cystine) (Thermo, 21013024). The third ferroptosis panel was combined RSL-3 (MCE, HY-100218A) with or without fer-1, DFO, Z-VAD, 3-MA, and nec-1s.

### Next-generation sequencing (NGS) and Gene Set Enrichment Analysis (GSEA)

The parental PANC-1 cell line and PANC-1 treated with cystine depletion were harvested for RNA extraction in triplicate (only alive adherent cells were collected for the RNA extraction). The samples were sent to the BGI Corporation (Shenzhen, China) for next-generation sequencing via the BGISEQ-500 sequencer. The differentially expressed genes (DEGs), Gene Set Enrichment Analysis (GSEA), and transcription factors extraction were performed by Dr Tom from BGI (https://biosys.bgi.com/#/report/login). LncRNA and mRNA sequencing data are available in the NCBI Gene Expression Omnibus under accession number GSE216569.

### Plasmid construction and transformation

The pCDH-CMV-MCS-EF1-puro plasmid was used to clone the LINC01133 and CEBPB. DH5a competent cells were mixed with 100 ng plasmid DNA, then incubated on ice for 30 min. The heat shock was then applied for 90 s at 42 °C. The culture was then cooled for 2 min on ice before mixing with 1 mL LB broth. The culture was incubated for 1 h at 37 °C with 250 rpm shaking. The culture was disseminated on an LB agar plate with antibiotics as needed. At 37 °C, the spread plate was incubated overnight. Then pick the white colonies for the plasmids isolation. The extraction of the plasmids followed the instructions of the PureLink™ HiPure Plasmid Midiprep Kit. The Sanger sequencing was used to corroborate the correct sequences.

### Malondialdehyde (MDA) assay

A lipid peroxidation test kit (ab118970) bought from Abcam was used to determine the relative MDA concentration in cell lysates according to the manufacturer’s instructions. DMSO, erastin, and cystine depletion were used to treat cells seeded on a 10 cm plate (1 × 10^7^ cells per plate) for 24 h. Then these cells were homogenized on ice in 300 μL MDA lysis buffer with 3 μL BHT (100×), then centrifuged (13,000 g, 10 min) to remove insoluble material. Transfer 200 μL of the supernatant from each homogenized sample to a microcentrifuge tube. Then fill each vial with 600 μL of thiobarbituric acid (TBA) solution. Then the mixtures were incubated for 60 min at 95 °C. An MDA–TBA adduct was formed when MDA in the sample interacted with TBA. In an ice bath, chill samples to room temperature for 10 min. For analysis, pipette 200 μL from each reaction mixture into a 96-well plate. Using a microplate reader (Bio-Rad), determine the absorbance at 532 nm.

### Cell proliferation assay

Approximately 3000 cells were seeded in the 96-well 24 h before the treatment for different times. A total of 10 μL CCK8 solution mixed with the medium was used to replace the medium of each well, followed by 1–4 h incubation after the treatment. The medium was then transferred to another 96-well plate. The absorbance was measured at 450 nm in the plate reader (Bio-Rad).

### Statistical analysis

GraphPad Prism 9 (GraphPad Software, La Jolla, CA) was harnessed for statistical analysis. Statistical methods in this project included a two-tailed Student *t*-test, a one-way ANOVA test followed by multiple comparisons with Bonferroni correction, a two-way ANOVA test, and an IC50 calculation. The data of each group were shown as mean ± standard deviation. *P* values less than 0.05 were regarded as statistically significant. All authors had access to the study data and had reviewed and approved the final manuscript.

## Results

### LINC01133 was significantly upregulated when ferroptosis was induced in the pancreatic cancer cell lines

**Fig. 1:**
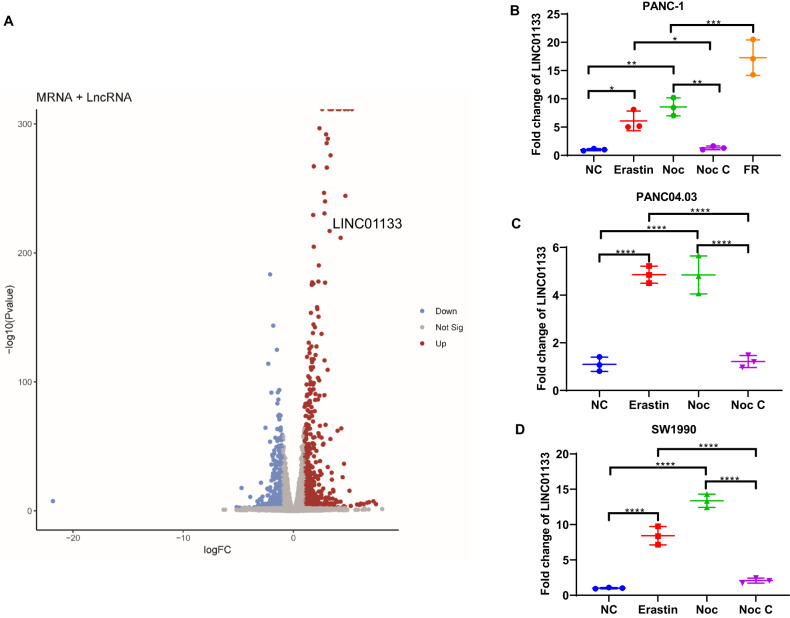
The LINC01133 expression changed the most significantly after ferroptosis induction. **A** The panc-1 treated with cystine depletion for 24 h and the control group in triplicates were used for the mRNA and lncRNA sequencing. A total of 1105 upregulated transcripts (red pots) and 699 downregulated transcripts (blue plots) were screened (|Fold Change| >2, *p* < 0.05). **B** LINC01133 expression in PANC-1 NC, PANC-1 Erastin, PANC-1 Noc, PANC-1 Noc C, and FR. **C** LINC01133 expression in panc 04. 03 NC, PANC-1 Erastin, PANC-1 Noc, PANC-1 Noc C, and FR. **D** LINC01133 expression in SW1990 NC, PANC-1 Erastin, PANC-1 Noc, PANC-1 Noc C, and FR. The NC group means the cell lines were cultured in a standard medium. The erastin group implies that the erastin for PANC-1 and PANC04.03 was 10 µM, and SW1900 was 2.5 µM for 24 h. The Noc group means the cell lines were treated with cystine starvation for 24 h. The NOC C group suggests that the three cell lines were treated with cystine starvation for 12 h, followed by 12-h standard culturing. FR means ferroptosis-resistant PANC-1 cultured in cystine-depleted DMEM for 24 h. One-way ANOVA was used for the statistical analysis. NS means *p* > 0.05, *p < 0.05, **p < 0.01, ***p < 0.001, ****p < 0.0001. All experiments were repeated three times independently.

To identify the critical non-coding RNAs in ferroptosis, we aimed to find the best model for our next-generation sequencing first. We treated pancreatic cancer cell lines in our lab, such as PANC-1, PANC04.03, SW1990, CFPAC-1, and Capan-2, with the well-recognized ferroptosis inducer erastin. The result indicated that except for CFPAC-1, all other four cell lines were susceptible to ferroptosis (Supplementary Fig. S1A). Next, we co-treat these cell lines with the erastin and dimethyl sulfoxide (DMSO) (1%), the ferroptosis inhibitor ferrostatin-1 (fer-1), the iron chelator deferoxamine (DFO), the apoptosis inhibitor Z-VAD-FMK (Z-VAD), autophagy inhibitor 3-methyladenine (3-MA) and necroptosis inhibitor necrostatin-1s (nec-1s). We found that in the four ferroptosis-sensitive pancreatic cancer cell lines PANC-1, PANC04.03, SW1990, and CAPAN-2, the inhibition by erastin could only be significantly rescued by the fer-1, DFO rather than Z-VAD or nec-1s. While the 3-MA had a negligible effect on the erastin treatment, the extent is not as great as the ferroptosis inhibitors (Supplementary Fig. S1B–E). Consistent with the sensitivity to ferroptosis, the inhibition of erastin to CFPAC-1 could not be reversed by any of the PCD inhibitors (Supplementary Fig. S1F). Since autophagy inhibitor 3-MA manifested different effects in these cell lines, we also treated three cell lines cultured in DMEM with another method to induce ferroptosis, i.e., cystine depletion. Likewise, cystine depletion was also used in combination with different PCD inhibitors. Because for these five cell lines, only modified DMEM could be bought from the market. We treated PANC-1, PANC04.03, and SW1990 with the cystine depletion combination. Consistently, the ferroptosis induced by cystine depletion was only rescued by the ferroptosis inhibitors (Supplementary Fig. S2A–C). Another ferroptosis inducer, GPX4 inhibitor RSL3, was also used to find the most suitable sequencing model. However, the rescuing effect was less significant than the erastin and cystine depletion (Supplementary Fig. S2D). Finally, we chose the PANC-1 treated with normal DMEM (control group) and cystine depletion (treatment group) for 24 h in triplicates as our next-generation sequencing model. For the mRNA and lncRNA sequencing, we obtained a total of 1105 upregulated transcripts and 699 downregulated transcripts (|Fold Change| >2, *p* < 0.05) (Fig. 1A). And the heatmap showed the DEG could differentiate the control and treatment groups (Supplementary Fig. S3). The gene set enrichment analysis (GSEA) of the mRNA sequencing data showed that ferroptosis was highly enriched (ranked 3rd) in the treatment group, with the normalized enrichment score as high as 2.49 (Supplementary Fig. S4A, B).

To further screen the potential candidates from the sequencing data, we set up a group of methods to treat the cancer cell lines, that is, to treat PANC-1/SW1990/PANC 0403 with the normal DMEM, erastin, cystine depletion, and cystine depletion (the first half of 24 h) + cystine supplementing (the second half of 24 h) for 24 h. The morphology of these cells undergoing ferroptosis differed from the control and rescue groups (Supplementary Fig. S2E). Through RNA expression detection of these cells with the treatment mentioned above by qRT-PCR, we found that LOC105378936, LINC01133, LINC02806, NUPR1, RND1, BGIG9606_51662, and ULBP1 here were upregulated more than 2-fold significantly (*p* < 0.05) when ferroptosis was induced in the three cell lines (Fig. 1B–D and Supplementary Fig. S5). More importantly, the seven transcripts decreased to the level of the control group in the cystine depletion + cystine supplementing group (Fig. 1B–D and Supplementary Fig. S5). During this period, one of our seven candidates, NUPR1, was published to be involved in ferroptosis, which further indicates our result is compelling [18, 19]. For the other seven candidates, we chose the LINC01133 as our prime target since the sequence-verified lncRNA changed the most significantly but with no relevant research on its correlation with ferroptosis. To further corroborate the role of LINC01133 in ferroptosis, a ferroptosis-resistant PANC-1 (PANC-1 FR) was established (the verification of resistance was described in the next part), and the level LINC01133 of this cell line was even higher than the PANC-1 treated with erastin or cystine depletion (Fig. 1B–D). And expression level of LINC01133 in the pancreatic cancer cell line was correlated with its sensitivity to ferroptosis (Supplementary Figs. S1A and S2F). These data implied that these transcripts especially LINC01133, might play an essential role in regulating ferroptosis.

### Upregulation of LINC01133 could induce resistance to ferroptosis in PAAD

**Fig. 2:**
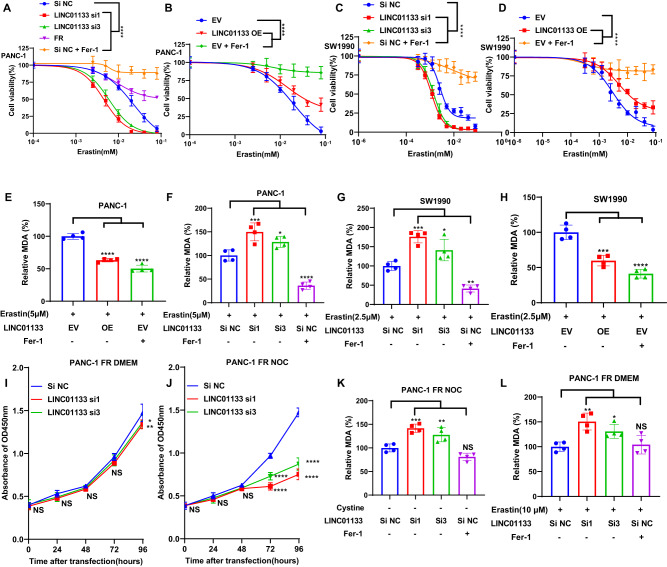
The effect of LINC01133 on ferroptosis sensitivity. The cell viability of PANC-1 (**A**) and SW1990 (**C**) treated with erastin of different concentrations for 24 h after LINC01133 knockdown. The cell viability of PANC-1 (**B**) and SW1990 (**D**) treated with erastin of different concentrations for 24 h after LINC01133 overexpression. The MDA level of PANC-1 treated with 5 µM erastin for 24 h after LINC01133 knockdown (**E**) and overexpression (**F**). The MDA level of SW1990 treated with 2.5 µM erastin for 24 h after LINC01133 knockdown (**G**) and overexpression (**H**). The cell proliferation of PANC-1 FR culturing in DMEM (**I**) or treated with cystine starvation (**J**) after LINC01133 knockdown at different time points. The MDA level of PANC-1 FR treated with cystine starvation (**K**) or 10 µM erastin (**L**) for 24 h after LINC01133 knockdown. Si NC means the control siRNA. Si1, and si3 are two effective siRNAs targeting LINC01133. FR is the ferroptosis-resistant PANC-1. Si NC + Fer-1 implies that the cell line transfected with the si NC was co-treated with 2 µM ferrostatin-1 when treated with erastin of different concentrations for 24 h. EV means the empty plasmid. OE implies the LINC01133 overexpression. EV + Fer-1 implies that the cell line transfected with the empty plasmid was co-treated with 2 µM ferrostatin-1 when treated with erastin of different concentrations for 24 h. One-way ANOVA and two-way ANOVA were used for the statistical analysis. NS means *p* > 0.05, *p < 0.05, **p < 0.01, ***p < 0.001, ****p < 0.0001. All experiments were repeated three times independently.

After choosing the LINC01133 as our prime research non-coding RNA, we knocked down the LINC01133 in PANC-1 by siRNA. The knockdown efficiency was detected by qRT-PCR. The siRNA1 and siRNA3 were selected as their efficiency was higher than 50% (Supplementary Fig. S6A). Since some research has focused on the protumor effect of LINC01133 in PAAD, we first detected the cell proliferation change after the knockdown [20–22]. Consistent with this research, PANC-1, with the decrease of LINC01133, demonstrated a relatively lower proliferation rate (Supplementary Fig. S6B). Next, to test whether the LINC01133 impacts the ferroptosis, we set up four groups, PANC-1 treated with siRNA control, PANC-1 treated with LINC01133 siRNA si1, PANC-1 treated with LINC01133 siRNA si3, PANC-1 treated with Fer-1. These four groups were treated with erastin of different concentrations for 24 h. As we expected, the result indicated that the knockdown of LINC01133 could significantly and greatly make PANC-1 more sensitive to ferroptosis. PANC-1 treated with fer-1 manifested an inadequate response to erastin, further verifying that the decrease of LINC01133 could sensitize PANC-1 to ferroptosis (Fig. 2A). Then, overexpression was performed in this cell line. And the data showed the opposite effect with the knockdown of LINC01133 (Fig. 2B and Supplementary Fig. S6C, D). Furthermore, we also detected the peroxidized product of ferroptosis, MDA, in these groups. Knocking down or overexpressing LINC01133 in PANC-1 without the treatment of erastin did not lead to a significant change in MDA (Supplementary Fig. S6E, F). Cotreatment of erastin and knockdown of LINC01133 caused a substantial increase of ferroptosis marker MDA, while LINC01133 overexpression combined with erastin could decrease the MDA significantly like the Fer-1 group (Fig. 2E, F). These experiments were also performed in another PAAD cell line SW1990 with a similar result (Fig. 2C, D, G, H and Supplementary Fig. S7A–F).

More importantly, we established the PANC-1 FR by culturing PANC-1 with normal DMEM and cystine depletion alternately (each treatment for 1 week for a full 3 months). The resistance was verified by comparing the proliferation rate of the PANC-1 FR and the parental PANC-1 cultured in DMEM for three months. Whether by the erastin treatment of different concentrations or cystine depletion, the PANC-1 FR showed significantly higher viability, i.e., more than 50% viability in as high as 20 μM erastin and the ability to proliferate in DMEM with cystine depletion (Supplementary Fig. S6G, H). Due to the extremely high level of LINC01133 in PANC-1 FR, we performed a knockdown of LINC01133 in the PANC-1 FR. The viability of PANC-1 FR with the knockdown of LINC01133 manifested much lower viability in cystine depletion. Although the efficacy of LINC01133 knockdown was significant in panc1-FR cultured in normal DMEM, the impact was relatively minimal compared with that of cystine depletion (Fig. 2I, J and Supplementary Fig. S6I, J). In the normal DMEM, the MDA generated by the PANC-1 FR with LINC01133 knockdown did not change significantly (Supplementary Fig. S7G). However, in the cystine depletion or treatment by erastin, the knockdown of LINC01133 did increase the MDA level compared with the control group (Fig. 2K, L). Taken together, LINC01133 contributed to the acquired resistance to ferroptosis in pancreatic cancer cell lines.

### LINC01133 predicted an advanced stage, lower immune infiltration, and poor prognosis in pancreatic cancer

**Fig. 3:**
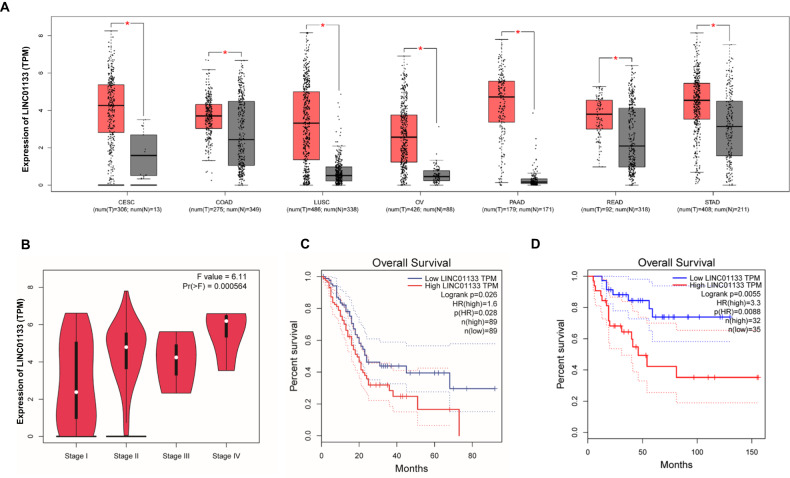
The effect of LINC01133 on clinical features. **A** The expression of LINC01133 between the tumor samples (T) and the adjacent normal tissues (N) in CESC, COAD, LUSC, OV, PAAD, READ, and STAD. **B** The expression of LINC01133 among different clinical stages of PAAD. **C**, **D** The prognosis of the high-LINC01133 and the low-LINC01133 group of PAAD (**C**) and ACC (**D**) in TCGA. CESC cervical squamous cell carcinoma and endocervical adenocarcinoma, COAD colon adenocarcinoma, LUSC lung squamous cell carcinoma, OV ovarian serous cystadenocarcinoma, PAAD pancreatic adenocarcinoma, READ rectum adenocarcinoma, STAD stomach adenocarcinoma, ACC adrenocortical carcinoma. The Student *t*-test, one-way ANOVA, and log-rank test were used for the statistical analysis. NS means *p* > 0.05, *p < 0.05, **p < 0.01, ***p < 0.001, ****p < 0.0001.

To better characterize the role of LINC01133, we took advantage of The Cancer Genome Atlas (TCGA) to its expression level. By using the online tool gene expression profiling interactive analysis (GEPIA) (http://gepia.cancer-pku.cn/), we found that in the cervical squamous cell carcinoma and endocervical adenocarcinoma (CESC), colon adenocarcinoma (COAD), lung squamous cell carcinoma (LUSC), ovarian serous cystadenocarcinoma (OV), pancreatic adenocarcinoma (PAAD), rectum adenocarcinoma (READ) and stomach adenocarcinoma (STAD), LINC01133 was significantly highly expressed in the tumor tissues compared with the normal tissues (Fig. 3A). Furthermore, in PAAD patients, a higher level of LINC01133 implied a higher clinical stage (Fig. 3B). The prognosis of PAAD was also worse in those patients with a higher level of LINC01133 (Fig. 3C). We also found that in adrenocortical carcinoma (ACC), higher expression of LINC01133 suggested a worse survival (Fig. 3D). And the immune score evaluation by the TIMER algorithm indicated that between the patients with a higher and a lower level of LINC01133, the CD8+ T cells and macrophages infiltration were significantly lower in the high-LINC01133 group (Supplementary Fig. S8A). Collectively, a high level of LINC01133 is associated with an advanced clinical stage, lower immune infiltration, and poor prognosis in pancreatic cancer.

### Higher expression of LINC01133 was associated with higher resistance to ferroptosis inducer in cancer

To find more robust evidence for the linkage of the LINC01133 and ferroptosis resistance, we also used the largest publicly available pharmacogenomics database (the Genomics of Drug Sensitivity in Cancer (GDSC), https://www.cancerrxgene.org/) to explore the LINC01133 expression and the response to sorafenib, an FDA-approved drug that was reported to be able to induce ferroptosis. To our surprise, not just in PAAD, there was a significantly higher half-maximal inhibitory concentration (IC50) of sorafenib in the LINC01133 highly expressed group compared with the low-LINC01133 group in 13 types of cancer (PAAD, breast invasive carcinoma (BRCA), CESC, cholangiocarcinoma (CHOL), COAD + READ, uterine corpus endometrial carcinoma (UCEC), STAD, mesothelioma (MESO), prostate adenocarcinoma (PRAD), kidney chromophobe (KICH) + kidney renal clear cell carcinoma (KIRC) + kidney renal papillary cell carcinoma (KIRP), testicular germ cell tumors (TGCT), thyroid carcinoma (THCA), and bladder urothelial carcinoma (BLCA)) (Supplementary Figs. S8B, C and S9A–L). Furthermore, we also searched all the available microarray and sequencing data associated with cancer and ferroptosis inducers from the gene expression omnibus (GEO) database. In GSE104462, LINC01133 was found to be upregulated by erastin in the liver cancer cell line HepG2 (Supplementary Fig. S10A). In two sorafenib-resistant liver cancer lines and one sulfasalazine-resistant oral squamous carcinoma cell line, the LINC01133 was also highly increased from GSE158458, GSE128683, and GSE113860 (Supplementary Fig. S10B–D). Altogether, it suggests that the linkage of LINC01133 and ferroptosis resistance is very general in various cancer.

### CEBPB was responsible for the upregulation of LINC01133 in ferroptosis resistance

**Fig. 4:**
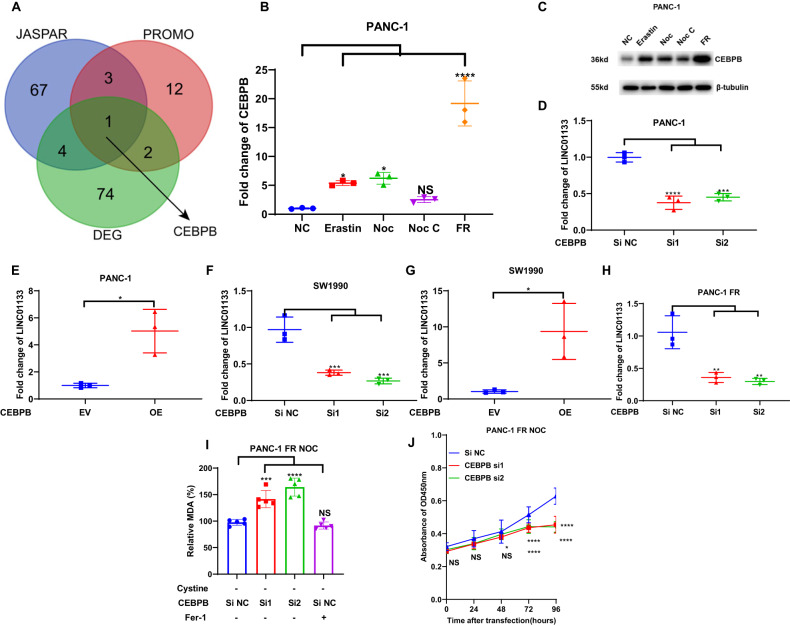
The regulation of CEBPB on LINC01133. **A** The overlapping gene among the differentially expressed RNA molecules from the sequencing, the predicted transcription factors by JSAPAR and PROMO. CEBPB mRNA level (**B**) and protein level (**C**) among PANC-1 NC, PANC-1 Erastin, PANC-1 Noc, PANC-1 Noc C, and PANC-1 FR. The change of LINC01133 expression after the CEBPB knockdown (**D**) or overexpression (**E**) in PANC-1. The change of LINC01133 expression after the CEBPB knockdown (**F**) or overexpression (**G**) in SW1990. **H** The change of LINC01133 expression after the CEBPB knockdown in PANC-1 FR. **I** The MDA level of PANC-1 FR treated with cystine starvation for 72 h after CEBPB knockdown. **J** The cell proliferation of PANC-1 FR treated with CEBPB knockdown and cyctine starvation. The NC group means the PANC-1 was cultured in a standard DMEM for 24 h. The erastin group implies that PANC-1 was treated with 10 µM erastin for 24 h. The Noc group means the cell line was treated with cystine starvation for 24 h. The Noc C group suggests that the cell line was treated with cystine starvation for 12 h, followed by 12-h standard culturing. FR means ferroptosis-resistant PANC-1 cultured in cystine-depleted DMEM for 24 h. Si NC means the control siRNA. Si1 and si2 are two effective siRNAs targeting CEBPB. EV means the empty plasmid. OE implies CEBPB overexpression. The Student *t*-test and one-way ANOVA were used for the statistical analysis. NS means *p* > 0.05, *p < 0.05, **p < 0.01, ***p < 0.001, ****p < 0.0001. All experiments were repeated three times independently except **A**.

**Fig. 5:**
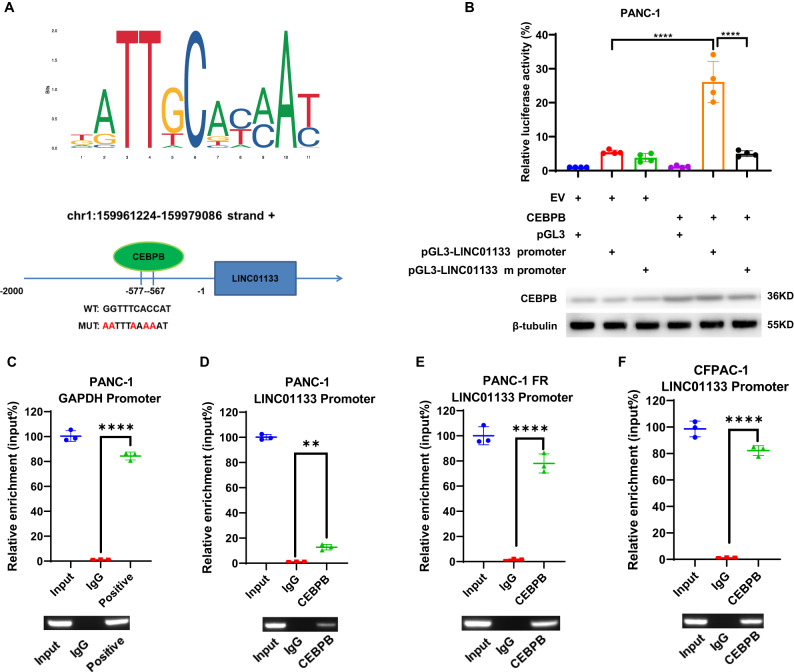
The binding of CEBPB to the promotor LINC01133. **A** The predicted binding site of the LINC01133’s promoter with CEBPB and its location in the promoter. **B** The relative luciferase activity change in PANC-1 transfected with EV + pGL3, EV + pGL3-LINC01133 promoter, EV + pGL3-LINC01133 mutant promoter, CEBPB + pGL3, CEBPB + pGL3-LINC01133 promoter, and CEBPB + pGL3-LINC01133 mutant promoter. The overexpression detection of CEBPB was detected by the western blot below. **C** The anti-RNA Polymerase II – GAPDH promoter binding served as the positive control. The enrichment of LINC01133 promoter by CEBPB in PANC-1 (**D**), PANC-1 FR (**E**), and CFPAC-1 (**F**). EV means the empty plasmid. The Student *t*-test and one-way ANOVA were used for the statistical analysis. NS means *p* > 0.05, *p < 0.05, **p < 0.01, ***p < 0.001, ****p < 0.0001. All experiments were repeated three times independently.

To determine how LINC01133 was upregulated during the ferroptosis resistance, we extracted the transcription factors from the DEG in our sequencing data by Dr Tom. Two transcription factor prediction tools, JASPAR and PROMO, were also harnessed to obtain the possible responsible transcription factors of the promoter region in the genomic location of LINC01133 (from –2000 to 100 nucleotides). By overlapping the DEG transcription factors and the two predictive transcription factor lists, we finally got the CEBPB (Fig. 4A). Firstly, we detected the change of the CEBPB in PANC-1 after erastin or cystine depletion treatment. The result by qRT-PCR demonstrated a consistent change with the LINC01133. Just like LINC01133, the expression of CEBPB returned to near the control level in the cystine depletion (the first half of 24 h) + cystine supplementing (the second half of 24 h) group, and the CEBEP increased to an extremely high level in the PANC-1 FR (Fig. 4B). And the protein level by immunoblotting also suggested a similar change to the RNA level (Fig. 4C). Then, we knocked down the CEBPB by siRNA in PANC-1, which was proven by the qRT-PCR and western blot (Supplementary Fig. S11A, C). Compared with the control group, the decrease of CEBPB could decrease LINC01133 (Fig. 4D). We also overexpressed the CEBPB in this cell line, followed by qRT-PCR and western blot corroboration (Supplementary Fig. S11B, F). As we expected, the qRT-PCR assay implied that the overexpression of CEBPB could lead to a raised level of LINC01133 (Fig. 4E). Then, we verified our hypothesis in another PAAD cell line SW1990, and similar results were obtained (Fig. 4F, G and Supplementary Fig. S11C–F). Consistently, the knockdown of CEBPB in PANC-1 FR could also lead to a decrease of LINC01133 (Fig. 4H and Supplementary Fig. S11G, H). Knockdown of CEBPB could also resensitize the PANC-1 FR to cystine depletion with higher MDA levels (Fig. 4I, J). Since the binding site predicted by JASPAR was GGTTTCACCAT, we mutated the binding site to the AATTTAAAAAT. Through dual-luciferase reporter assay, it showed that mutation of the predictive binding site could significantly lower the transcription of the luciferase compared with the WT promoter (Fig. 5A, B). The chromatin immunoprecipitation (CHIP) assay suggested that CEBPB could bind to the promoter of the LINC01133 in PANC-1, PANC-1 FR, and CFPAC-1 (Fig. 5C–F). More interestingly, a higher expression of CEBPB could indicate a higher sorafenib resistance in 12 types of cancer in GDSC (liver hepatocellular carcinoma (LIHC), BRCA, CESC, COAD + READ, UCEC, STAD, MESO, PRAD, KICH + KIRC + KIRP, TGCT, THCA, and BLCA) (Supplementary Figs. S10D, E and S12A–L). And CEBPB was positively correlated with LINC01133 in PAAD (Supplementary Fig. S8G). Together, we could conclude that CEBPB was responsible for the upregulation of LINC01133 in the acquired resistance to ferroptosis.

### LINC01133 could regulate the FSP1 expression rather than DHODH and GPX4

**Fig. 6:**
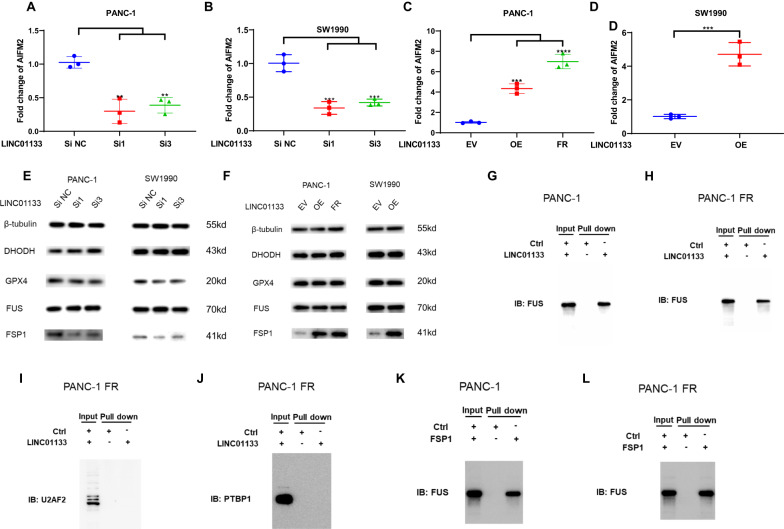
The validation of the LINC01133-FUS-FSP1 complex. The AIFM2 mRNA expression change after LINC01133 knockdown in PANC-1 (**A**) and SW1990 (**B**). The AIFM2 mRNA expression change after LINC01133 overexpression in PANC-1, PANC-1 FR (**C**) and after LINC01133 overexpression in SW1990 (**D**). **E** GPX4, DHODH, AIFM2, FUS and beta-tubulin protein levels in PANC-1 and SW1990 after LINC01133 knockdown. **F** GPX4, DHODH, AIFM2, FUS and beta-tubulin protein levels in PANC-1 and SW1990 after LINC01133 overexpression and PANC-1 FR. The FUS was pulled down by LINC01133 in PANC-1 (**G**) and PANC-1 FR (**H**). The U2AF2 (**I**) or PTBP1 (**J**) was not pulled down by LINC01133 in PANC-1 FR. The FUS was pulled down by FSP1 mRNA in PANC-1 (**K**) and PANC-1 FR (**L**). Si NC means the control siRNA. Si1 and si3 are two effective siRNAs targeting LINC01133. EV means the empty plasmid. OE implies the LINC01133 overexpression. FR means the ferroptosis-resistant PANC-1 cultured in cystine-depletion medium for 24 h. The Student *t*-test and one-way ANOVA were used for the statistical analysis. NS means *p* > 0.05, *p < 0.05, **p < 0.01, ***p < 0.001, ****p < 0.0001. All experiments were repeated three times independently.

After finding the molecules that control the transcription of LINC01133, we continued to find how LINC01133 could lead to acquired ferroptosis. After knocking down this transcript in PANC-1, we detected the main antioxidant molecules that could prevent ferroptosis, such as GPX4, FSP1, and DHODH. We found that the decrease of the LINC01133 could cause the downregulation of the FSP1 mRNA rather than GPX4 and DHODH in both PANC-1 and SW1990 (Fig. 6A, B and Supplementary Fig. S13A–D). Conversely, overexpression of this non-coding RNA could make the mRNA level of FSP1 increase significantly without affecting the status of the GPX4 and DHODH (Fig. 6C, D and Supplementary Fig. S13E–H). The protein level of FSP1 was also detected by immunoblotting after knocking down or overexpressing this lncRNA. Our data implicated that the protein level had a consistent change with the mRNA level. Only FSP1 increased when LINC01133 was overexpressed rather than GPX4 or DHODH, and the protein of FSP1 decreased after the knockdown of the LINC01133 (Fig. 6E, F). And FSP1 was positively correlated with LINC01133 in PAAD (Supplementary Fig. S8H). Based on these data, it can be concluded that the acquired resistance to ferroptosis by LINC01133 increase may be associated with its regulation on FSP1.

### LINC01133-FUS-FSP1 complex could enhance the FSP1 stability to lead to ferroptosis resistance

**Fig. 7:**
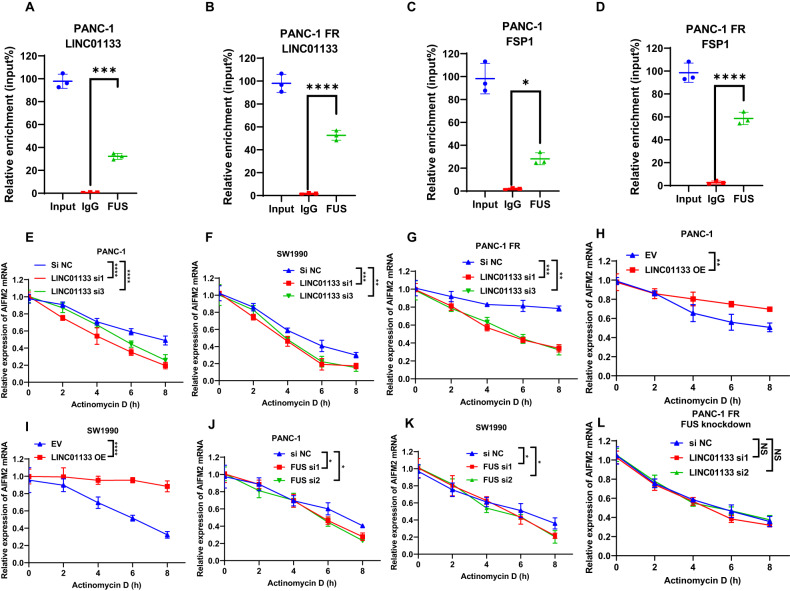
The stability of AIFM2/FSP1 mRNA regulated by LINC01133 and FUS. The relative enrichment of LINC01133 by FUS in PANC-1 (**A**) and PANC-1 FR (**B**). The relative enrichment of FSP1 by FUS in PANC-1 (**C**) and PANC-1 FR (**D**). The relative expression of FSP1 at different time points of 10 μg/mL actinomycin D treatment after LINC01133 knockdown in PANC-1 (**E**), SW1990 (**F**), and PANC-1 FR (**G**). The relative expression of FSP1 at different time points of 10 μg/mL actinomycin D treatment after LINC01133 overexpression in PANC-1 (**H**), SW1990 (**I**). The relative expression of FSP1 mRNA at different time points of 10 μg/mL actinomycin D treatment after FUS knockdown in PANC-1 (**J**) and SW1990 (**K**). **L** The relative expression of FSP1 mRNA at different time points of 10 μg/mL actinomycin D treatment after LINC01133 knockdown in PANC-1 FR with FUS knockdown. The Student *t*-test and the two-way ANOVA were used for the statistical analysis. NS means *p* > 0.05, *p < 0.05, **p < 0.01, ***p < 0.001, ****p < 0.0001. All experiments were repeated three times independently.

**Fig. 8:**
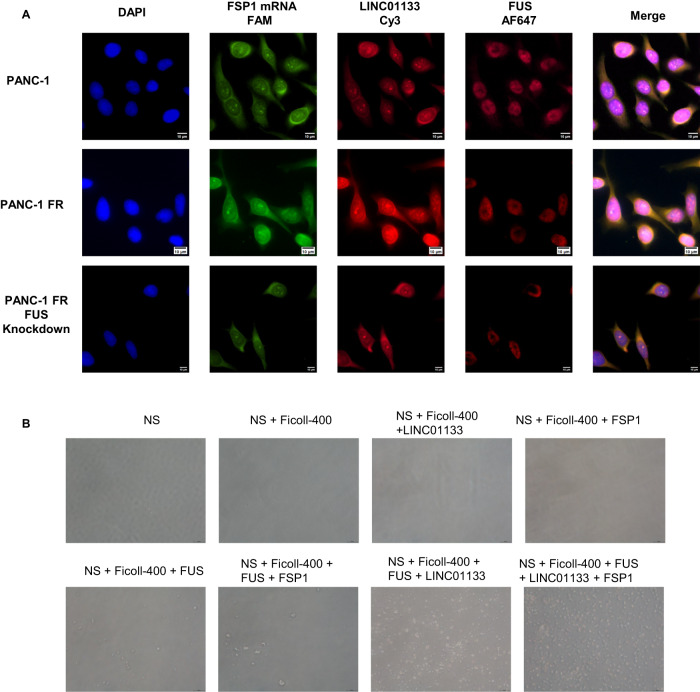
The locolization and phase separation of LINC01133, FSP1 and FUS. **A** The location of LINC01133 (Cy3 label), FSP1 mRNA (FAM label) and FUS protein (AF647) by FISH and IF in panc-1, panc-1 FR, and panc-1 FR with FUS knockdown. In the merge picture, AF647 was labeled magenta with LINC01133 and FSP1 the same color. **B** The image of the phase separation of LINC01133, FUS and FSP1 mRNA. 300 ng/μL LINC01133 or FSP1 mRNA was used. For the FUS, 16 μM final concentration was chosen. The reaction was in the normal saline supplemented with 12.5% Ficoll-400.

To further identify how FSP1 was regulated by the LINC01133, we first use bioinformatic tools to predict whether there was a proximate interaction between the FSP1 mRNA and LINC01133. However, the analyses by the Lnctar (http://www.cuilab.cn/lnctar) and LncRRIsearch (http://rtools.cbrc.jp/LncRRIsearch/index.cgi) ruled out the possible interaction between the two transcripts (Supplementary Fig. S14A, B). Besides, the only RNA that could bind to the LINC01133 predicted by ENCORI (https://starbase.sysu.edu.cn/) was AC005332.5 (Supplementary Fig. S14C). So, this made us think about whether there was a mediator involving the LINC01133’s regulation on FSP1. ENCORI was also used to predict the potential proteins that could bind to the LINC01133. Finally, a list of a total of 13 proteins was obtained. The top three of them were PTBP1, FUS, and U2AF2 (Supplementary Fig. S14D). In order to find which protein could interact with the LINC01133, we in vitro transcribe the LINC01133. Our RNA pull-down assay indicated that only FUS could bind to the LINC01133 rather than PTBP1 or U2AF2 (Fig. 6G–J). The LINC01133 knockdown or overexpression did not affect the expression of FUS (Fig. 6E, F and Supplementary Fig. S13I–L). Interestingly, the ENCORI suggested that FUS could bind to FSP1/AIFM2 (Supplementary Fig. S14E). Then, we in vitro transcribed the FSP1 mRNA. The RNA pull-down assay by FSP1 mRNA validated the binding between FSP1 and FUS (Fig. 6K, L). Furthermore, the RNA immunoprecipitation (RIP) experiment also showed that FUS could bind to both FSP1 and LINC01133 (Fig. 7A–D). The highly consistent location of the LINC01133, FUS, and FSP1 by FISH and IF corroborated the existence of the triple complex (Fig. 8A). More importantly, the colocalization was less evident after FUS was knocked down in PANC-1 FR (Fig. 8A). We hypothesized that the complex might affect the mRNA stability of FSP1, for instance, decreasing its degradation. After the actinomycin D treatment, the FSP1 decreased fast in PANC-1 or SW1990 treated with LINC01133 siRNA (Fig. 7E, F). This phenomenon was more evident in PANC-1 FR after LINC01133 knockdown (Fig. 7G). Overexpression of LINC01133 could make the decrease of FSP1 slower (Fig. 7H, I). The FUS knockdown could also promote the degradation of FSP1 (Fig. 7J, K). In the PANC-1 FR cell line, the LINC01133 knockdown lost its protective effect on the stability of the mRNA of FSP1 (Fig. 7L). More importantly, the LINC01133 can significantly increase FUS’s phase separation in vitro (Fig. 8B).

## Discussion

Ferroptosis has been validated as a modality of cell death distinct from apoptosis and has shown promising anti-tumor effects, especially for apoptosis-inducers-resistant tumors [23–25]. A study showed that in gastric cancer cells of the mesenchymal state, the expression of elongation of very-long-chain fatty acid protein 5 (ELOVL5) and fatty acid desaturase 1 (FADS1) was upregulated, resulting in ferroptosis sensitization, which was opposite in intestinal-type gastric cancer cells [26]. In breast cancer, metformin coupled with sulfasalazine, a glutamate/cystine antiporter inhibitor, can promote ferroptosis and synergistically decrease breast cancer cell growth [27]. In pancreatic cancer, a combination of ferroptosis inducers and apoptosis inducers greatly increased the lethal impact of gemcitabine [28]. Despite the fact that the mechanism of ferroptosis was gradually uncovered, the crucial non-coding RNAs related to ferroptosis still need more exploration. In this study, we found LOC105378936, LINC01133, LINC02806, BGIG9606_51662 that have not been reported to be associated with ferroptosis manifested a significant ferroptosis-induced change. These lncRNAs were significantly increased in three PAAD cell lines by two different ferroptosis inducers. They could return to nearly normal if the cell lines were rescued by cystine supplementation. More intriguingly, NUPR1, an RNA molecule we selected as a candidate, had been published to participate in ferroptosis [18]. This indicated that our candidates are up-and-coming in the regulation of ferroptosis. However, some candidates still need more evidence to validate their relation to ferroptosis. Collectively, we discovered some crucial RNA molecules that could play a role in ferroptosis.

LncRNAs have been confirmed to involve nearly every hallmark of cancer [29–31]. LINC01133 was also reported to participate in the development of various types of cancer. Previous research has found that LINC01133 could inhibit gastric cancer by sponging miR-576-5p [32]. While in pancreatic, cervical, and renal carcinoma, pioneer work suggested that LINC01133 could promote their proliferation [22, 33, 34]. Interestingly, another researcher found that LINC01133 could encourage the development of gastric cancer [35]. LINC01133 was also involved in the epithelial-mesenchymal transition through exosome release [36]. However, there is a lack of research on LINC01133 and ferroptosis. In the current study, we discovered that LINC01133 could increase the resistance to ferroptosis in pancreatic cancer. This lncRNA was even higher in our established ferroptosis-resistant PAAD cell line than in the group treated with ferroptosis inducers. More importantly, the knockdown of LINC01133 could reverse the acquired ferroptosis resistance. A higher LINC01133 meant a higher IC50 of sorafenib, which could induce ferroptosis in many cancers. What is intriguing is that we did not see the IC50 difference in liver cancer (Supplementary Fig. S8C), while the sequencing data in GSE158458 and GSE128683 agreed with our result. That may be explained by the complicated environment of liver cancer. More clinical data was needed to understand this. Besides, sorafenib could also induce apoptosis, autophagy, and cell cycle arrest, but we could only obtain the IC50 of this ferroptosis inducer in the clinic database GDSC. Although ferroptosis provides us with an alternative to cancer therapy, the acquired resistance to it could be expected in the future. Thus, we uncovered a pathway by which the cancer cells obtained resistance to ferroptosis and provided a method to overcome this process.

Transcription factors are a general regulator for the transcription of many genes. Various types of cancer were validated to be associated with the transcription factors. CEBPB was a well-studied transcription factor and had also been found to participate in cancer development. It has been reported that CEBPB could contribute to initiating hormone receptor-negative breast cancer by upregulating CLDN1 and LCN2 [37]. The result from another study proved that one isoform of CEBPB could directly induce PD-L1 transcription when non-small cell lung cancer lines were treated with metformin [38]. CEBPB could also promote the transcription of SLC16A3 to help the progression of ovarian cancer [39]. A previous record indicated that CEBPB could regulate the liver’s circadian clock by affecting the hepatic transcription [40]. However, the role of CEBPB in ferroptosis resistance still needs more research. In this study, we confirmed that CEBPB was the main transcription factor leading to increased LINC01133. The CEBPB’s level is much higher in the ferroptosis-resistant PANC-1. And higher CEBPB group was also found to have a higher IC50 of sorafenib. However, the clinical database indicated no significant difference in sorafenib sensitivity between high-CEBPB and low-CEBPB groups of pancreatic cancer (Supplementary Fig. S8D) and cholangiocarcinoma (Supplementary Fig. S12C). That may be on account of other effects of sorafenib and complicated environments in humans. And more research is needed to explain the phenomenon. Taken together, our result expanded the function of CEBPB and pinpointed another target to decrease the ferroptosis resistance.

The way the lncRNAs affect the downstream molecules is various. LncRNAs can alter RNA splicing, stability, and translation by interacting with DNA, RNA, and proteins. They can also control chromatin structure and function, as well as the transcription of nearby and distant genes. In addition, long non-coding RNAs have a role in the creation and control of organelles and nuclear condensates. In chromatin regulation, chromatin de-compaction could be induced by lncRNA on account of the opposite charge [41]. The protein can sometimes act as a mediator to facilitate the interaction between lncRNA and chromatin [42]. For instance, lncRNA ANRIL could promote the transcription of the CDKN2A and CDKN2B genes by recruiting PRC1 and PRC2 to their promoters to regulate senescence [43]. And lncRNA was also found to be able to silence gene expression. For example, the lncRNA XIST has been confirmed to silence the X chromosome by changing its spatial structure [44, 45].

Other mechanisms of how lncRNAs participate in the different biological processes include the miRNA sponge, RNA/DNA complex, scaffolding, coding peptide, etc. [46, 47]. Our study found that LINC01133 could increase the mRNA stability of FSP1 by forming the LINC01133-FUS-FSP1 complex. This complex was a triple complex that was seldom reported by other researchers. In the beginning, we tried to find whether there is a direct interaction between the LINC01133 and FSP1. The bioinformatic analysis by two tools ruled this out. Then, we hypothesized there might be a protein mediator involving the regulation. To our surprise, we finally obtained the three candidates. After RNA pull-down validation, we finally confirmed the existence of the LINC01133-FUS-FSP1 complex. The knockdown of LINC01133 or FUS could affect the FSP1 stability. The LINC01133 lost its protection on FSP1 after the FUS knockdown. These results made our hypothesis reasonable. However, we have to admit that more research is needed to explore the function of the triple-molecule complex. We could see the overlapping location of the triple complex by immunofluorescence (IF) and Fluorescence In Situ Hybridization (FISH). The overlapping was less evident after the FUS knockdown. Since the FUS has been reported to mediate phase separation in many biological processes [48–50], we further studied whether phase separation plays a role in protecting FSP1 stability. The mix of the LINC01133 with the FUS increased the triple complex’s phase separation, which might be the underlying mechanism. We will delve into this aspect in our future research.

## Conclusions

**Fig. 9:**
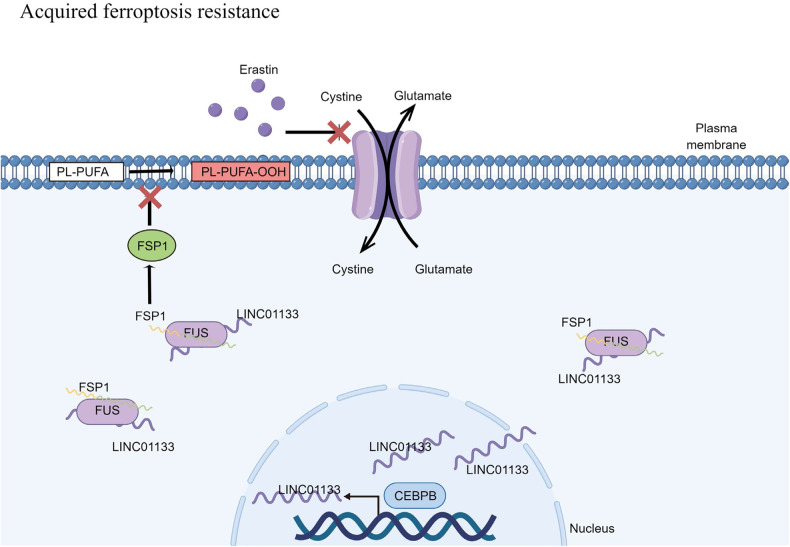
The mechanism of acquired ferroptosis resistance. The LINC01133 regulated by CEBPB can make pancreatic cancer cells more resistant to ferroptosis by enhancing the mRNA stability of FSP1 through forming the LINC01133-FUS-FSP1 complex.

In summary, we uncovered the differentially expressed non-coding RNA molecules after ferroptosis induction. We further selected seven candidates not reported in ferroptosis through validation in three cell lines treated with two ways of ferroptosis induction. Notably, we further confirmed that the increase of LINC01133 could decrease the sensitivity to ferroptosis in two PAAD cell lines. More importantly, we established a ferroptosis-resistant pancreatic cancer cell line and further validated the LINC01133’s function in the cell line. The decrease of LINC01133 could even reverse the ferroptosis resistance. Our experiment also demonstrated that the transcription factor CEBPB was the main criminal responsible for its upregulation by CHIP and dual-luciferase assay. We validated that LINC01133 could form a triple complex with FUS protein and FSP1 mRNA to enhance the stability of the potent ferroptosis suppressor, FSP1, to decrease the sensitivity to ferroptosis (Fig. 9 by figdraw).

### Reporting summary

Further information on research design is available in the Nature Research Reporting Summary linked to this article.

### Supplementary information

Supplementary material-figures

Supplementary material-WB uncropped

Reporting Summary

## Data Availability

LncRNA and mRNA sequencing data are available in the NCBI Gene Expression Omnibus under accession number GSE216569. The original data within the paper will be available from the lead contact upon request. The uncropped western blotting figures are included in the Supplementary materials. This paper does not report the original code. Any additional information in this paper is available from the lead contact upon request.
