# Supplementary material for: LINC01133 can induce acquired ferroptosis resistance by enhancing the FSP1 mRNA stability through forming the LINC01133-FUS-FSP1 complex
Source: Cell Death Dis. 2023 Nov 25;14(11):767. doi: 10.1038/s41419-023-06311-z (PMC10676390; doi:10.1038/s41419-023-06311-z)
Supplement: Supplementary file 1 — Supplementary material-figures [file 41419_2023_6311_MOESM1_ESM.pdf]

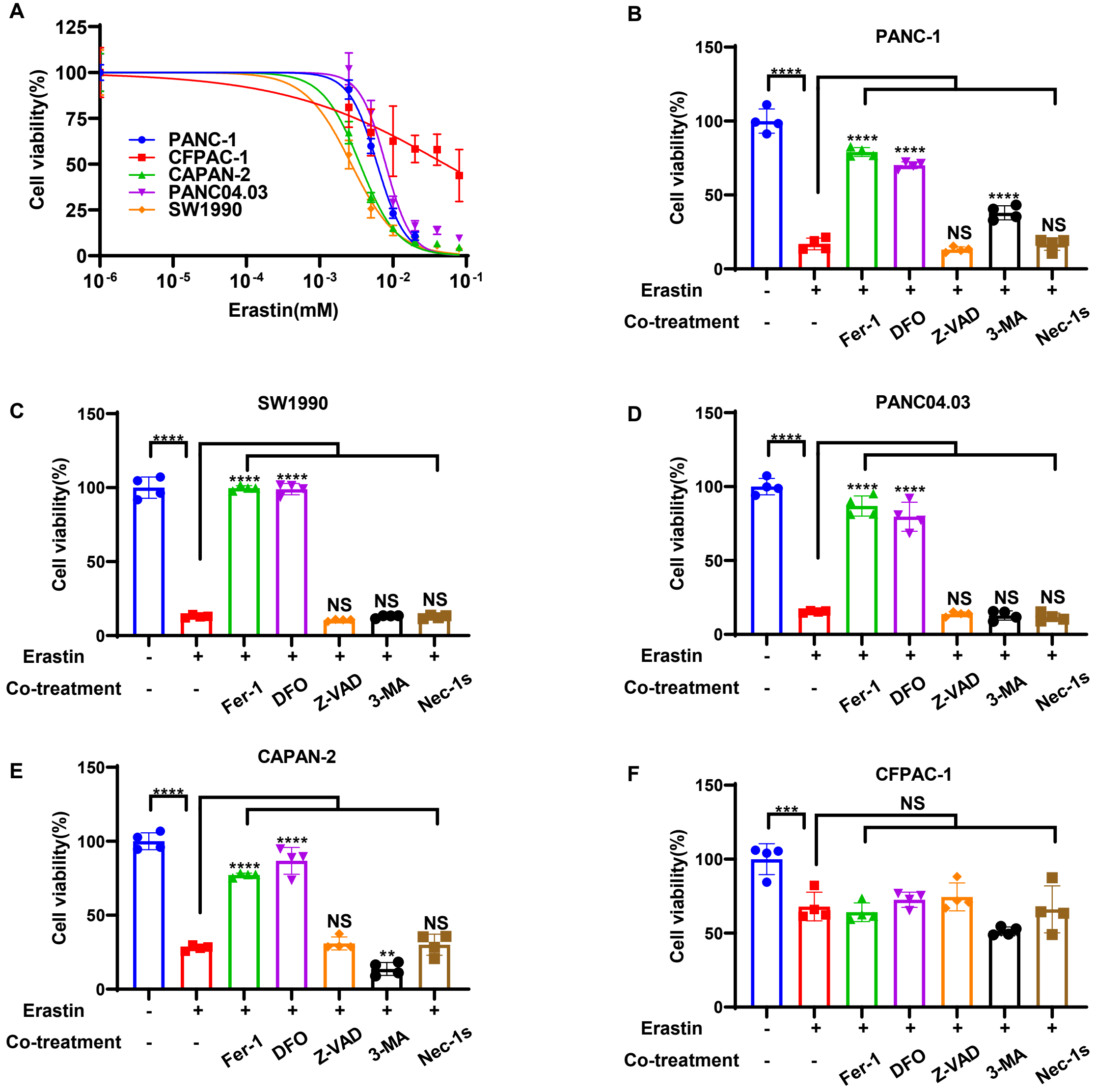

Figure S1. Cell viability of PAAD cell lines treated with an erastin ferroptosis panel.

(**A**) 3,000/well panc-1, panc 04.03, sw 1990, cfpac-1, and capan-2 in a 96-well plate were treated with erastin of different concentrations for 24 hours.
(**B-F**) 3,000/well panc-1, sw 1990, panc 04.03, capan-2, and cfpac-1 in a 96-well plate were treated with erastin in combination with 2 μM ferrostatin-1 (fer-1), 100 μM deferoxamine (DFO), 20 μM Z-VAD-FMK (Z-VAD), 2 mM 3-methyladenine (3-MA), or 2 μM necrostatin-1s (nec-1s). The viability was measured by CCK-8. The concentration of erastin for panc-1, panc 04.03, and capan-2 is 10μM, sw1990 is 2.5μM, and cfpac-1 is 20μM.
(One-way ANOVA was used for the statistical analysis. NS means $p > 0.05$, * means $P < 0.05$, ** means $P < 0.01$, *** means $P < 0.001$, **** means $P < 0.0001$. All experiments were repeated four times independently.)

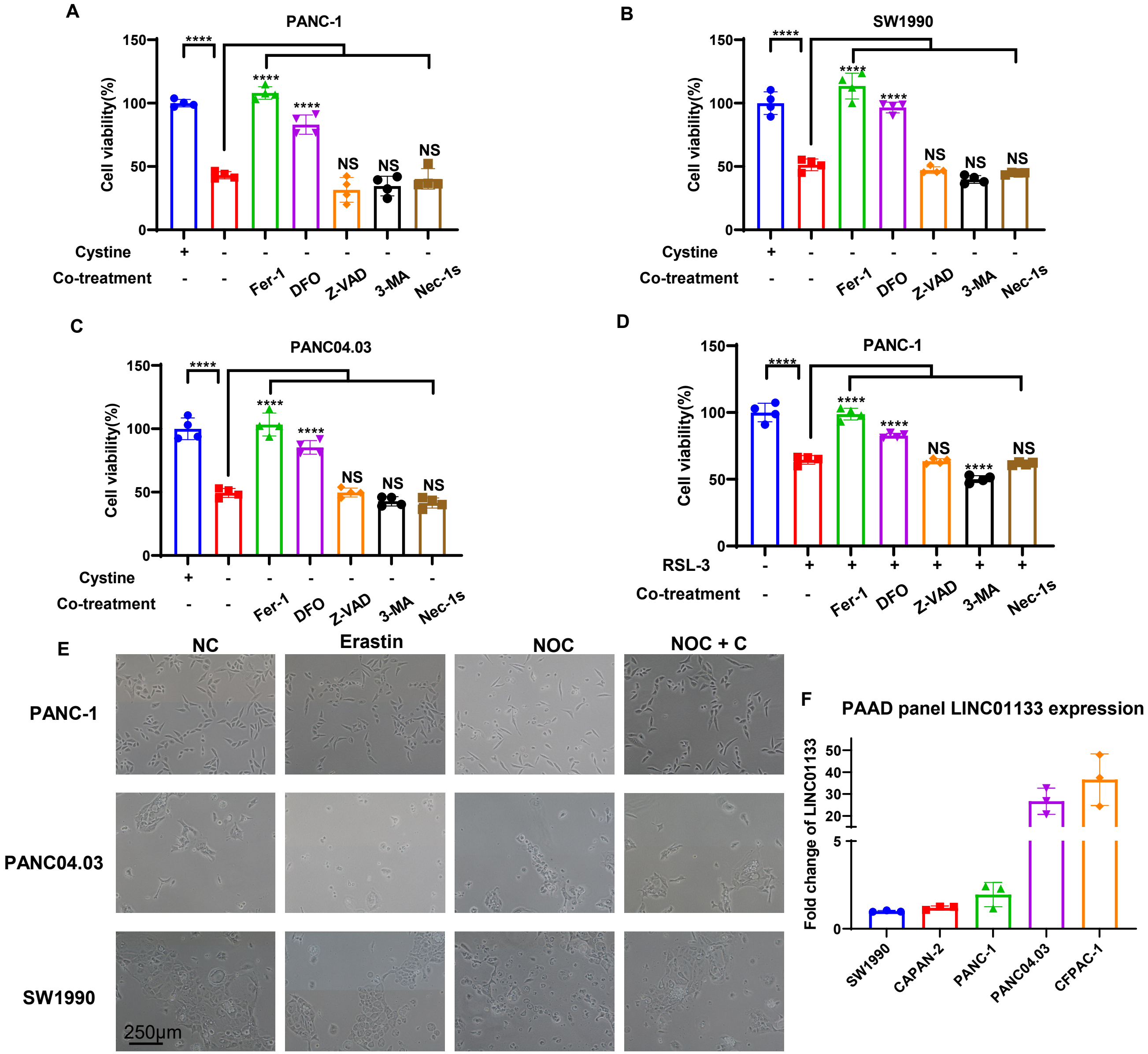

Figure S2. Cell viability of PAAD cell lines treated with a cystine depletion ferroptosis panel and RSL-3 ferroptosis panel.

(**A-C**) 3,000/well panc-1, sw 1990, and panc 04.03 in a 96-well plate were treated with cystine in combination with 2 μM ferrostatin-1 (fer-1), 100 μM deferoxamine (DFO), 20 μM Z-VAD-FMK (Z-VAD), 2 mM 3-methyladenine (3-MA), or 2 μM necrostatin-1s (nec-1s) for 24 hours.

(**D**) 3,000/well panc-1 in a 96-well plate was treated with 2.5 μM RSL-3 in combination with 2 μM ferrostatin-1 (fer-1), 100 μM deferoxamine (DFO), 20 μM Z-VAD-FMK (Z-VAD), 2 mM 3-methyladenine (3-MA), or 2 μM necrostatin-1s (nec-1s) for 24 hours. The viability was measured by CCK-8.

(E) The morphology of panc-1, panc 04.03, and sw1900 induced ferroptosis by virtue of two methods.

(F) The LINC01133 expression in pancreatic cancer cell lines.

(One-way ANOVA was used for the statistical analysis. NS means $p > 0.05$, * means $P < 0.05$, ** means $P < 0.01$, *** means $P < 0.001$, **** means $P < 0.0001$. All experiments were repeated three times independently.)

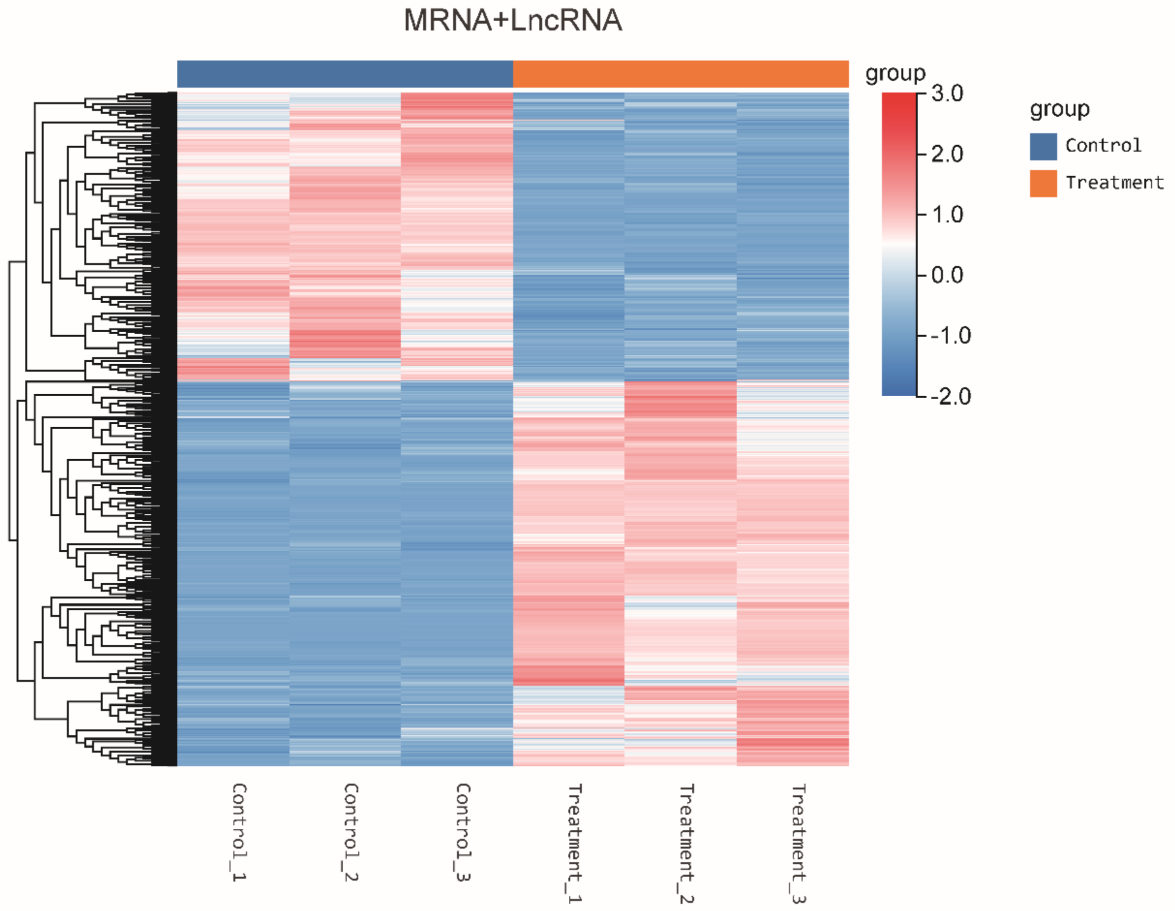

Figure S3. The heatmap of mRNA and lncRNA sequencing.

The differentially expressed transcripts could differentiate the control group and treatment group.

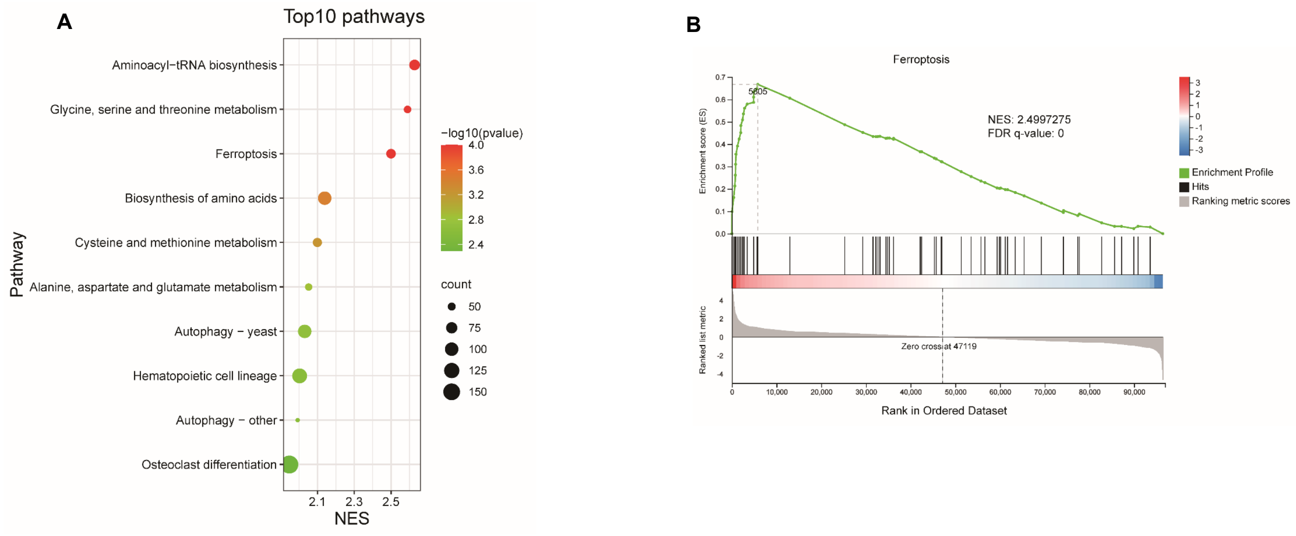

Figure S4. The GSEA analysis and the top 10 enriched pathways

(**A**) Of the top 10 pathways of the lncRNA and mRNA sequencing enriched by GSEA, ferroptosis ranked third.
(**B**) The GSEA analysis result of the ferroptosis pathway.
(NES means normalized enrichment score, FDR means false discovery rate.)

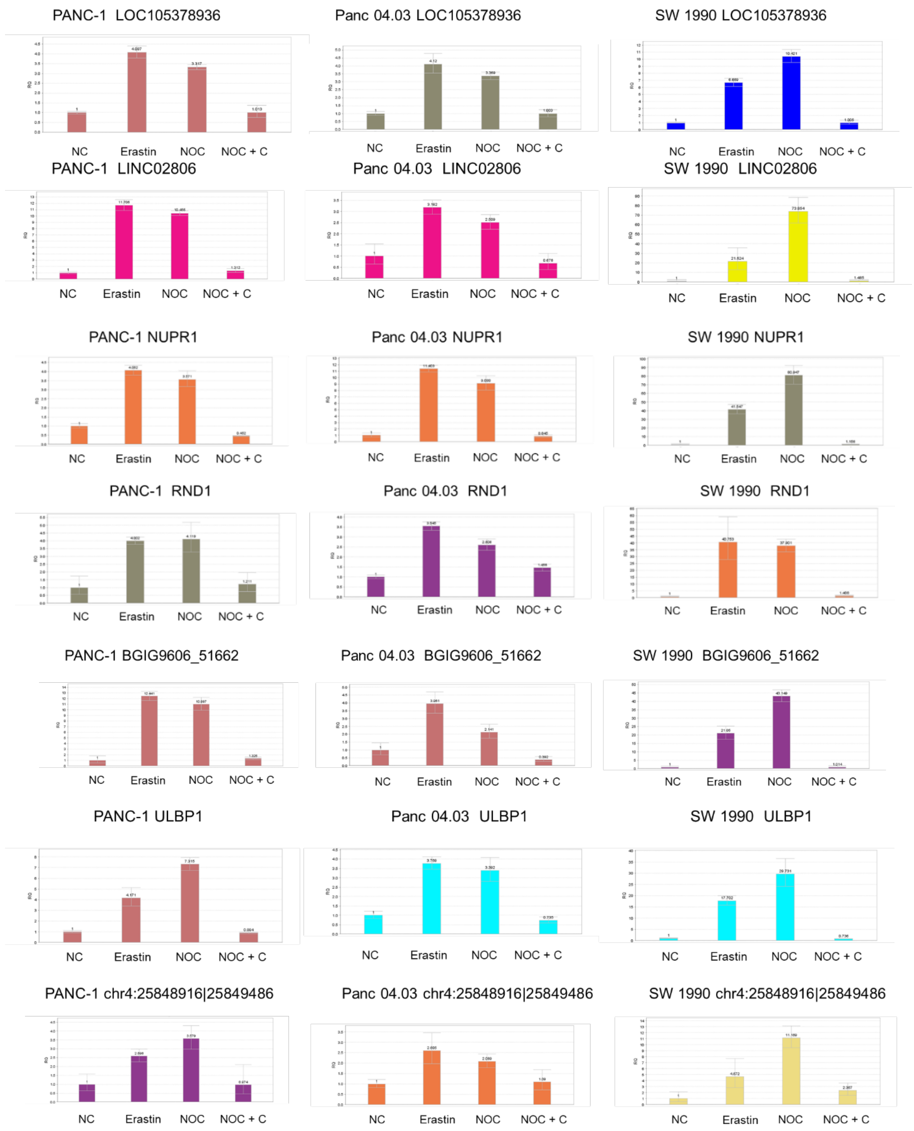

Figure S5. Candidate RNA molecules in ferroptosis regulation by qRT-PCR

The qRT-PCR results of LOC105378936, LINC02806, NUPR1, RND1, BGIG9606_51662, ULBP1, and circRNA(chr4:25848916|25849486) in panc-1, panc 04.03, and sw1900 induced ferroptosis by virtue of two methods.

(The NC group means the cell lines were cultured in a standard medium. The erastin group implies that the erastin for panc-1 and panc 04.03 was 10 μM, and sw 1900 was 2.5 μM for 24 hours. The NOC group means the cell lines were treated with cystine starvation for 24 hours. The NOC + C group suggests that the three cell lines were treated with cystine starvation for 12 hours, followed by 12-hour standard culturing. All experiments were repeated three times independently.))

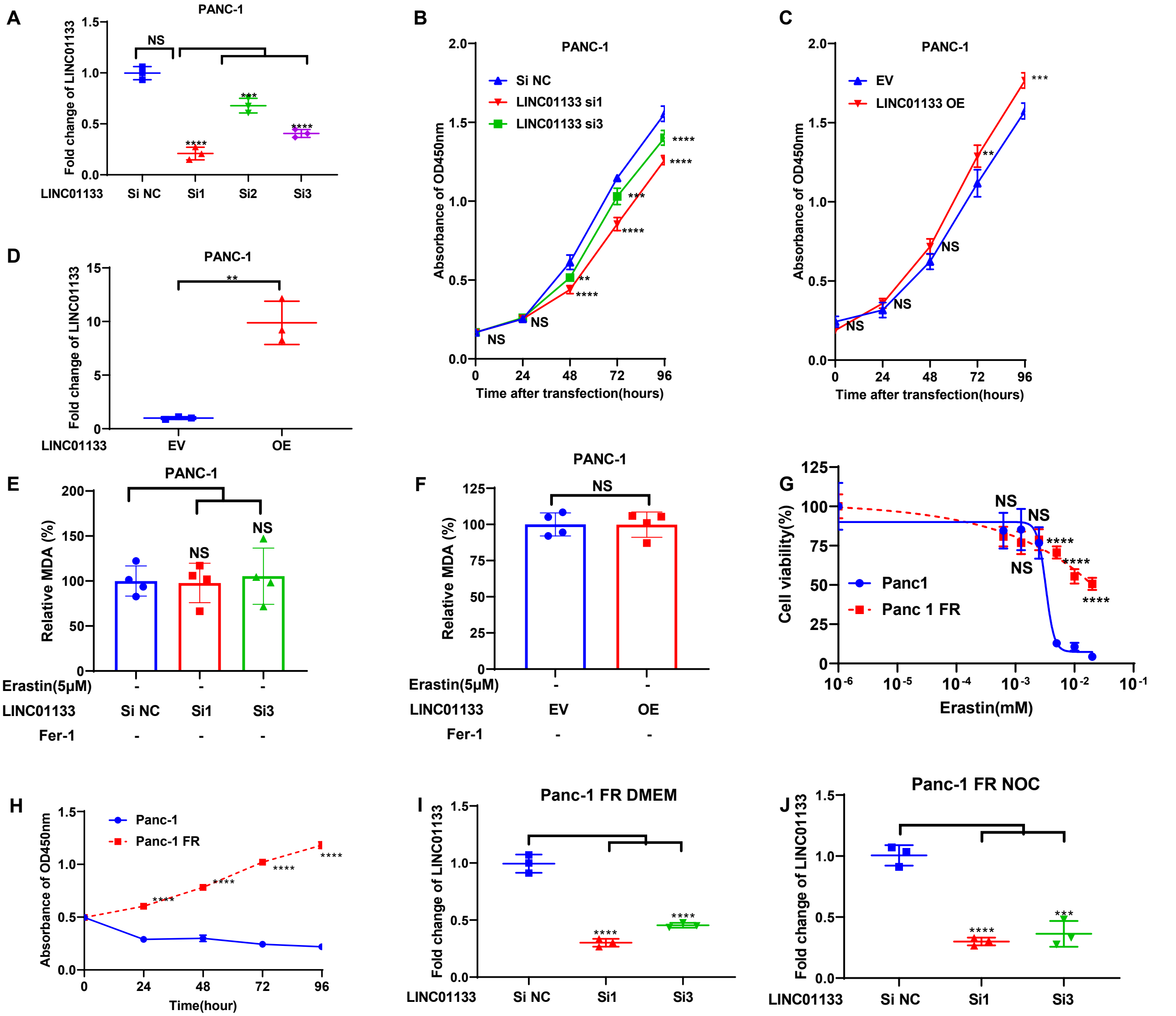

Figure S6. The validation of the knockdown and overexpression of LINC01133 in panc-1 and panc-1 FR.

(**A**) LINC01133 expression in panc-1 treated with three siRNAs of LINC01133 by qRT-PCR.
The proliferation curve of panc-1 after LINC01133 knockdown (**B**) or overexpression (**C**).
(**D**) LINC01133 expression in panc-1 transfected with the LINC01133 plasmid by qRT-PCR.
(**E**) The MDA level of panc-1 transfected with si NC, si1, and si3.
(**F**) The MDA level of panc-1 after LINC01133 overexpression.
(**G**) The cell viability of panc-1 and panc-1 FR under the treatment of erastin of different concentrations for 24 hours.
(**H**) The cell viability of panc-1 and panc-1 FR in the treatment of cystine depletion for 96 hours.
LINC01133 expression in Panc1 FR culturing in standard condition (**I**) or cystine-depleted DMEM (**J**) transfected with 2 effective siRNAs of LINC01133.
(Si NC means the control siRNA. Si1, si2, and si3 are three candidate siRNAs targeting LINC01133. EV means the empty plasmid. OE implies the LINC01133 overexpression. The student t-test, one-way ANOVA and two-way ANOVA were used for the statistical analysis. NS means p > 0.05, * means P < 0.05, ** means P < 0.01, *** means P < 0.001, **** means P < 0.0001. All experiments were repeated three times independently.)

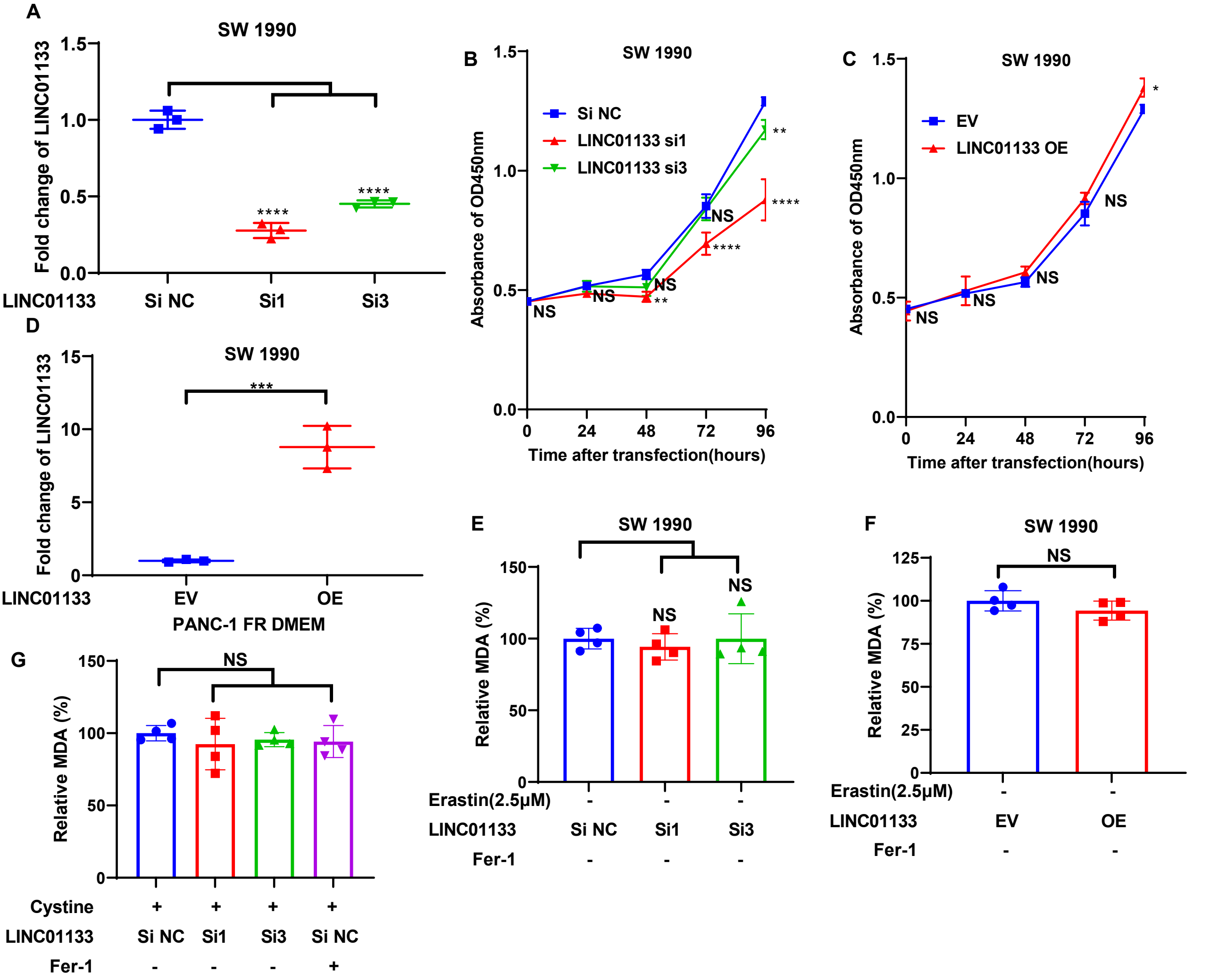

Figure S7. The validation of the knockdown and overexpression of LINC01133 in sw1990.

(**A**) LINC01133 expression in sw1990 treated with 2 effective siRNAs of LINC01133 by qRT-PCR.
The proliferation curve of sw1990 after LINC01133 knockdown (**B**) or overexpression (**D**).
(**C**) LINC01133 expression in sw1990 transfected with the LINC01133 plasmid by qRT-PCR.
The MDA level of sw1990 after LINC01133 knockdown (**E**) or overexpression (**F**).
(**G**) The MDA level of panc-1 FR transfected with si NC, si1, and si3.
(Si NC means the control siRNA. Si1 and si3 are two effective siRNAs targeting LINC01133. Si NC + Fer-1 implies that panc-1 FR transfected with the si NC was co-treated with 2 μM ferrostatin-1 when treated with cystine depletion or 10 μM erastin for 24 hours. The student t-test, one-way ANOVA and two-way ANOVA were used for the statistical analysis. NS means $p > 0.05$, * means $P < 0.05$, ** means $P < 0.01$, *** means $P < 0.001$, **** means $P < 0.0001$. All experiments were repeated three times independently.)

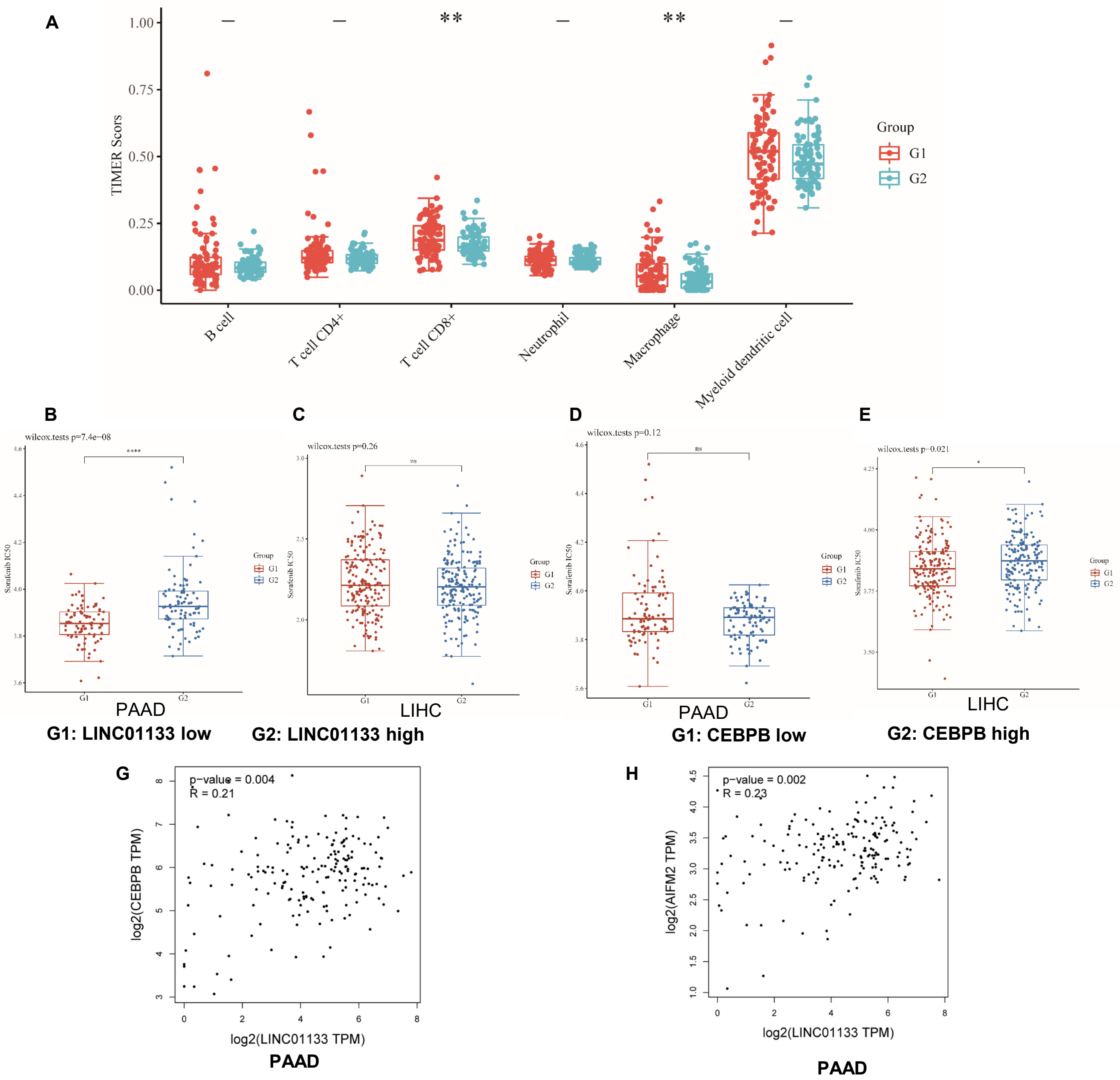

Figure S8. The effect of LINC01133 and CEBPB on the IC50 of sorafenib.

(**A**) The immune infiltration analysis of PAAD patients with high or low expression of LINC01133.
The IC50 between patients with low expression (G1) and high expression (G2) of LINC01133 in PAAD (**B**), LIHC (**C**).
The IC50 between patients with low expression (G1) and high expression (G2) of CEBPB in PAAD (**D**), LIHC (**E**).
(**G**) The correlation between CEBPB and LINC01133 in PAAD.
(**H**) The correlation between FSP1 and LINC01133 in PAAD.
(Pancreatic adenocarcinoma (PAAD), liver hepatocellular carcinoma (LIHC). Wilcox test and TIMER algorithm were used for the statistical analysis. NS means $p > 0.05$, * means $P < 0.05$, ** means $P < 0.01$, *** means $P < 0.001$, **** means $P < 0.0001$.)

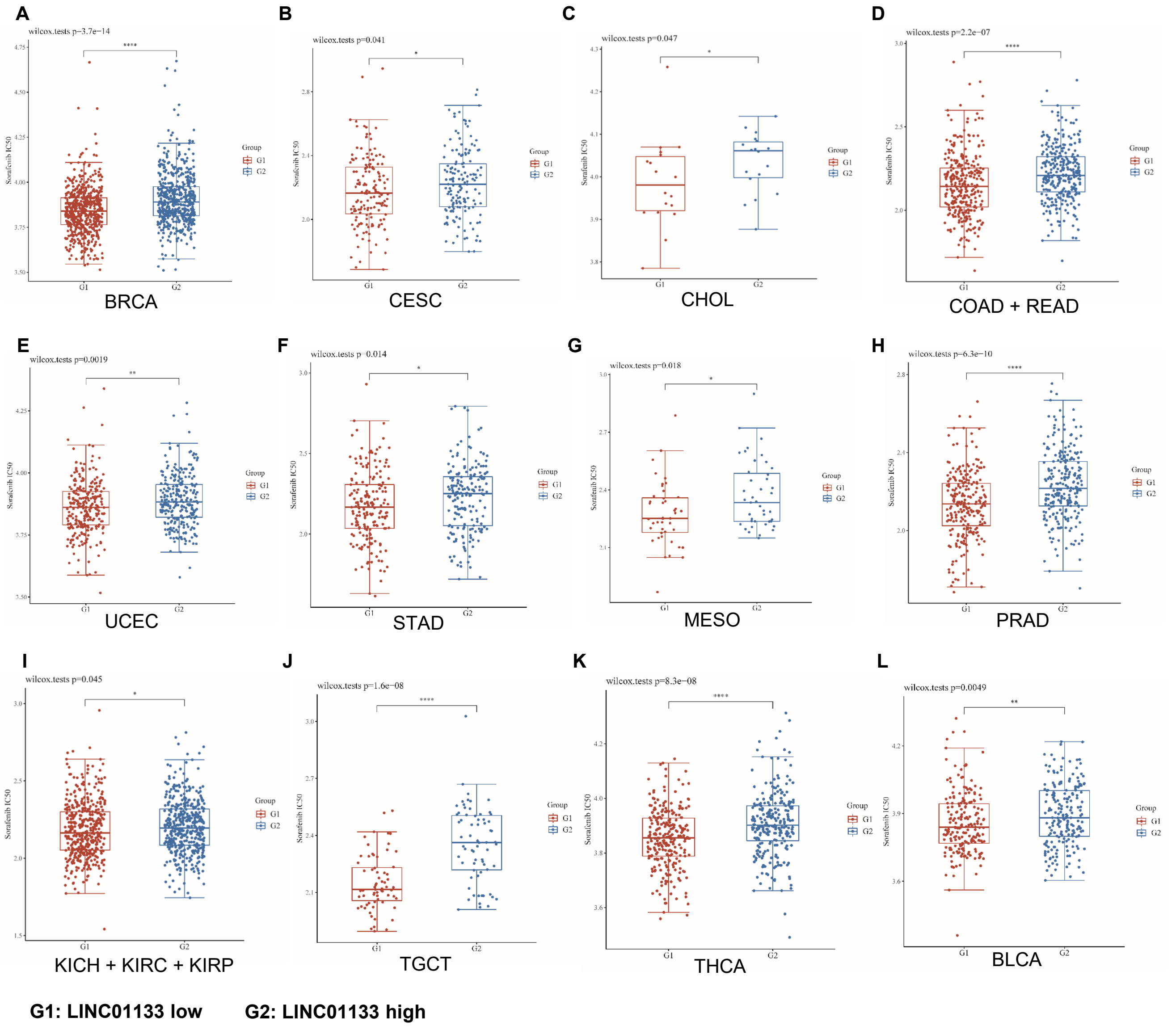

Figure S9. The effect of LINC01133 on the IC50 of sorafenib.

The IC50 between patients with low expression (G1) and high expression (G2) of LINC01133 in BRCA (**A**), CESC (**B**), CHOL (**C**), COAD + READ (**D**), UCEC (**E**), STAD (**F**), MESO (**G**), PRAD (**H**), KICH + KIRC + KIRP (**I**), TGCT (**J**), THCA (**K**), BLCA (**L**).
(Breast invasive carcinoma (BRCA), cervical squamous cell carcinoma and endocervical adenocarcinoma (CESC), cholangiocarcinoma (CHOL), colon adenocarcinoma (COAD) + rectum adenocarcinoma (READ), uterine corpus endometrial carcinoma (UCEC), stomach adenocarcinoma (STAD), mesothelioma (MESO), prostate adenocarcinoma (PRAD), kidney chromophobe (KICH) + kidney renal clear cell carcinoma (KIRC) + kidney renal papillary cell carcinoma (KIRP), testicular germ cell tumors (TGCT), thyroid carcinoma (THCA), and bladder urothelial carcinoma (BLCA). Wilcox test was used for the statistical analysis. NS means $p > 0.05$, * means $P < 0.05$, ** means $P < 0.01$, *** means $P < 0.001$, **** means $P < 0.0001$.)

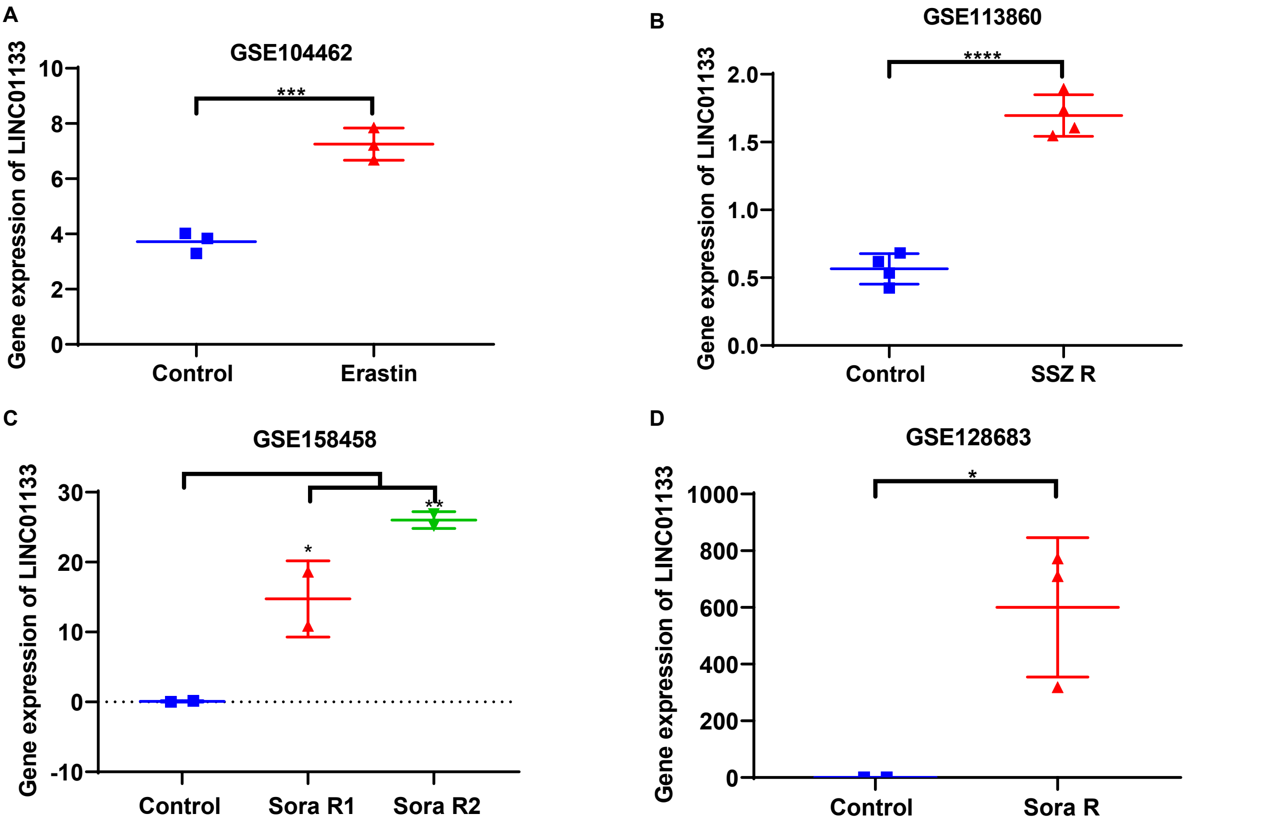

Figure S10. The expression of LINC01133 with ferroptosis induction in GEO database.

(**A**) LINC01133 expression between the HepG2 control group and the HepG2 treated with erastin from GSE104462.
(**B**) LINC01133 expression between the OCS19 control group and OCS19 resistant to sulfasalazine from GSE113860.
(**C**) LINC01133 expression between the Huh7 control group and the Huh7.IR and Huh7.CR resistant to sorafenib from GSE158458.
(**D**) LINC01133 expression between the HepG2 control group and the HepG2 resistant to sorafenib from GSE128683.
(The student t-test was used for the statistical analysis. NS means $p > 0.05$, * means $P < 0.05$, ** means $P < 0.01$, *** means $P < 0.001$, **** means $P < 0.0001$.)

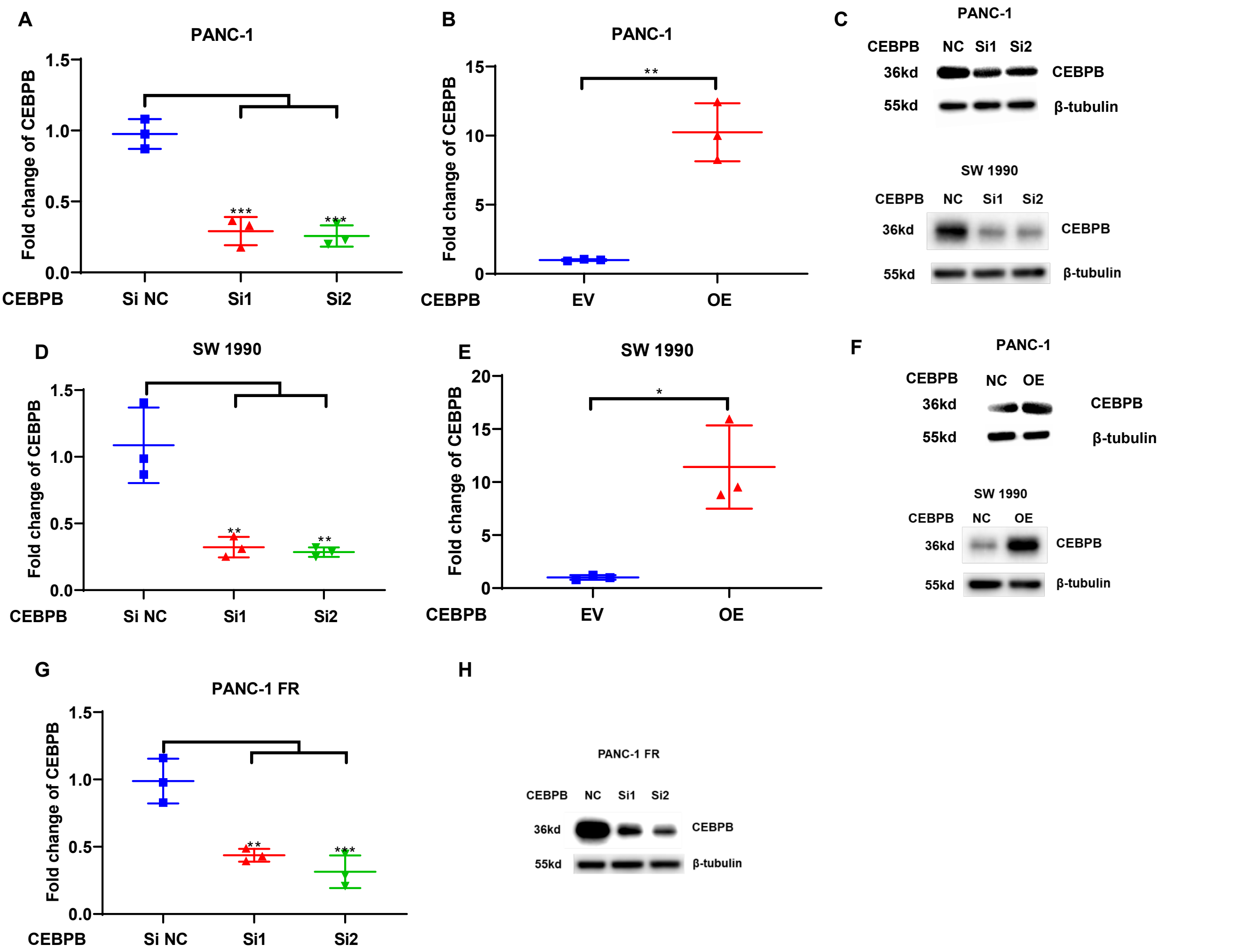

Figure S11. The validation of the knockdown and overexpression of CEBPB.

CEBPB mRNA levels' change after a knockdown by 2 siRNAs in panc-1 (**A**), sw1990 (**D**), and panc-1 FR (**G**).
CEBPB mRNA levels' change after an overexpression by its plasmid transfection in panc-1 (**B**) and sw1990 (**E**).
(**C**) CEBPB protein levels' change after its knockdown in panc-1 and sw1990.
(**F**) CEBPB protein levels'' change after an overexpression by its plasmid transfection in panc-1 and sw1990.
(**H**) CEBPB protein levels' change after its knockdown in panc-1 FR.
(Si NC means the control siRNA. Si1 and si2 are two effective siRNAs targeting CEBPB. EV means the empty plasmid. OE implies the CEBPB overexpression. The student t-test and one-way ANOVA were used for the statistical analysis. NS means p > 0.05, * means P < 0.05, ** means P < 0.01, *** means P < 0.001, **** means P < 0.0001. All experiments were repeated three times independently.)

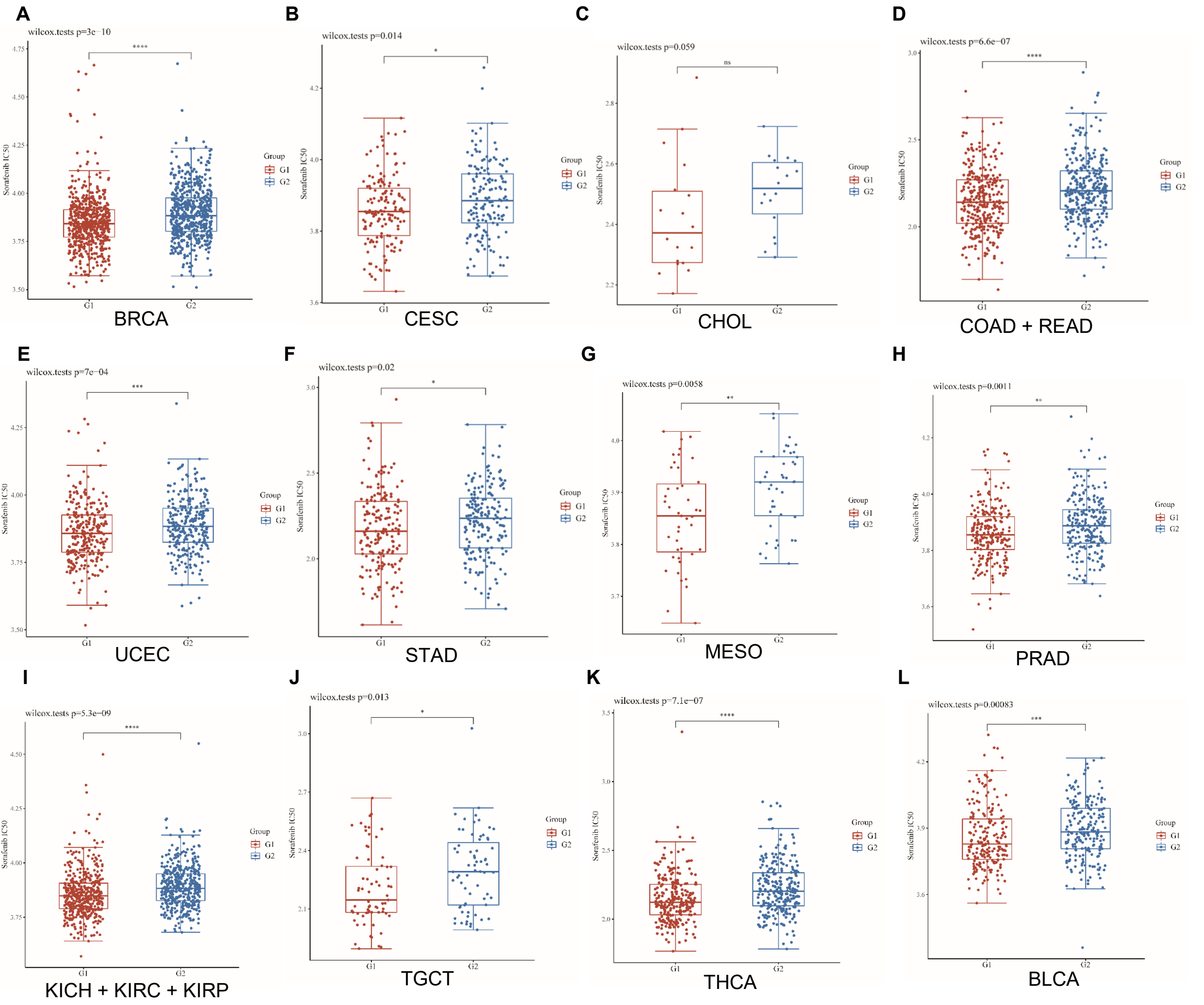

G1: CEBPB low **G2: CEBPB high**

Figure S12. The effect of CEBPB on the IC50 of sorafenib.

The IC50 between patients with low expression (G1) and high expression (G2) of CEBPB in BRCA (**A**), CESC (**B**), CHOL (**C**), COAD + READ (**D**), UCEC (**E**), STAD (**F**), MESO (**G**), PRAD (**H**), KICH + KIRC + KIRP (**I**), TGCT (**J**), THCA (**K**), BLCA (**L**).
(Breast invasive carcinoma (BRCA), cervical squamous cell carcinoma and endocervical adenocarcinoma (CESC), cholangiocarcinoma (CHOL), colon adenocarcinoma (COAD) + rectum adenocarcinoma (READ), uterine corpus endometrial carcinoma (UCEC), stomach adenocarcinoma (STAD), mesothelioma (MESO), prostate adenocarcinoma (PRAD), kidney chromophobe (KICH) + kidney renal clear cell carcinoma (KIRC) + kidney renal papillary cell carcinoma (KIRP), testicular germ cell tumors (TGCT), thyroid carcinoma (THCA), and bladder urothelial carcinoma (BLCA). Wilcox test was used for the statistical analysis. NS means $p > 0.05$, * means $P < 0.05$, ** means $P < 0.01$, *** means $P < 0.001$, **** means $P < 0.0001$.)

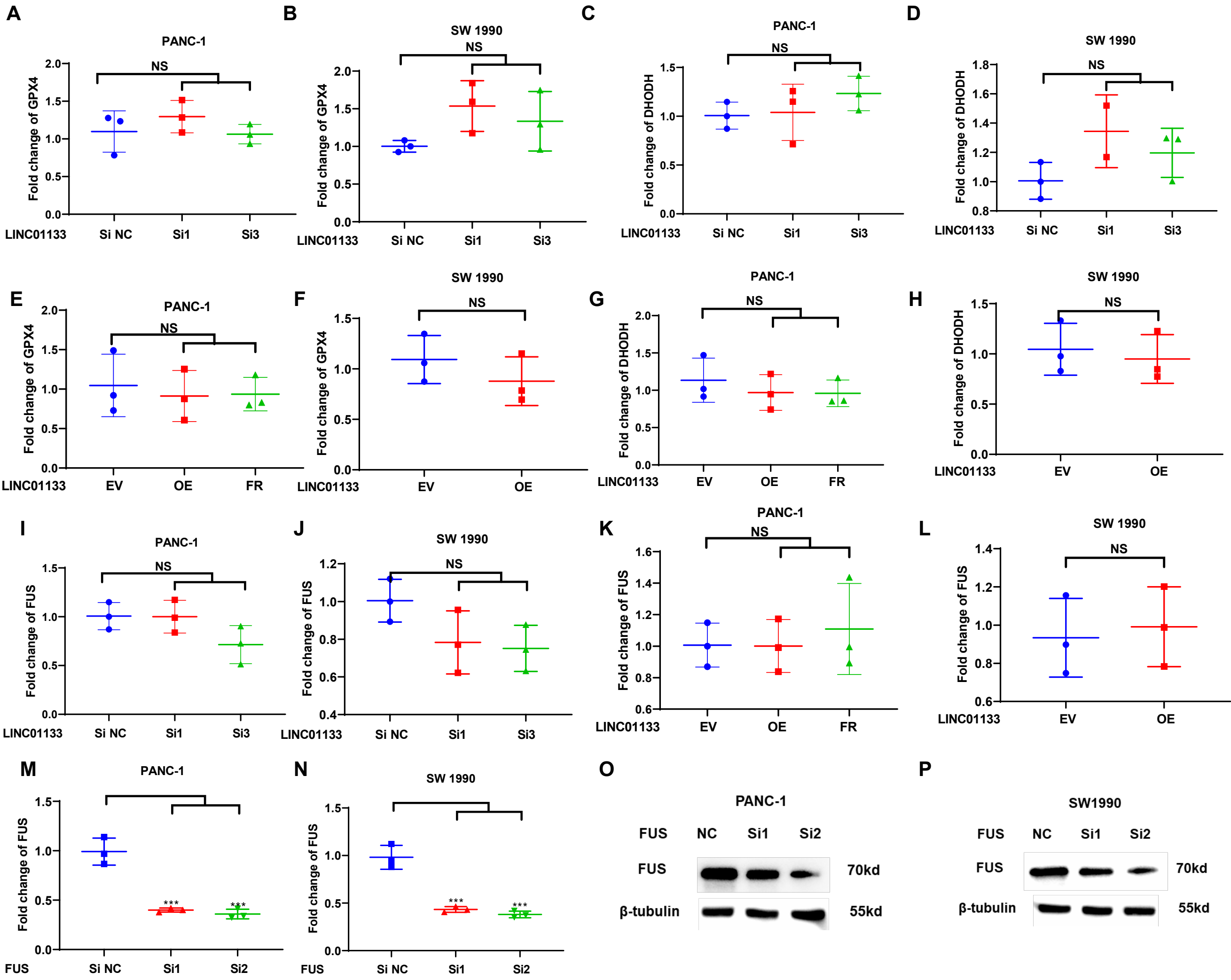

Figure S13. The vital antioxidant molecules' mRNA change by LINC01133 knockdown and the validation of FUS knockdown.

The GPX4 mRNA expression alteration after LINC01133 knockdown in panc-1 (**A**) and sw1990 (**B**).
The DHODH mRNA expression change after LINC01133 knockdown in panc-1 (**C**) and sw1990 (**D**).
The GPX4 mRNA expression alteration after LINC01133 overexpression in panc-1, panc-1 FR (**E**) and after LINC01133 overexpression in sw1990 (**F**).
The DHODH mRNA expression change after LINC01133 overexpression in panc-1, panc-1 FR (**G**) and after LINC01133 overexpression in sw1990 (**H**).
The FUS mRNA expression change after LINC01133 knockdown in panc-1 (**I**) and sw1990 (**J**).
The FUS mRNA expression change after LINC01133 overexpression in panc-1, panc-1 FR (**K**) and after LINC01133 overexpression in sw1990 (**L**).
The mRNA level of FUS in panc-1 (**M**) and sw1990 (**N**) after FUS siRNAs transfection.
The protein level of FUS in panc-1 (**O**) and sw1990 (**P**) after FUS siRNAs transfection
(The student t-test and one-way ANOVA were used for the statistical analysis. NS means $p > 0.05$, * means $P < 0.05$, ** means $P < 0.01$, *** means $P < 0.001$, **** means $P < 0.0001$. All experiments were repeated three times independently.)

A

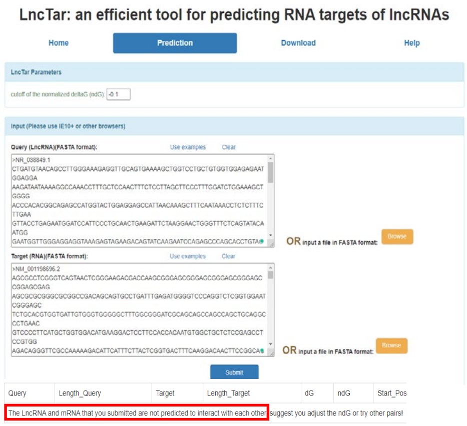

B

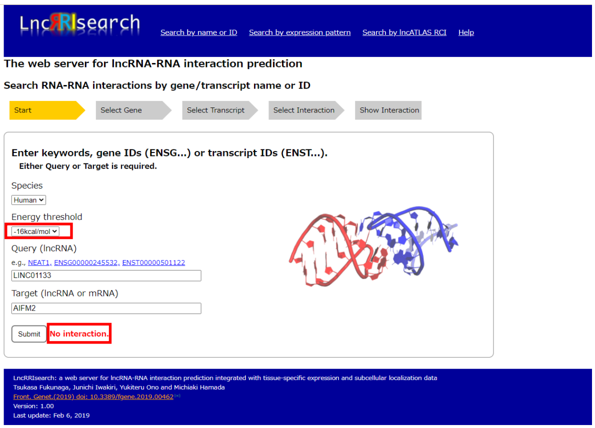

C

GeneID	GeneName	GeneType	PairGeneID	PairGeneName	PairGeneType	Interactions	ExpNum	SeqTypeNum	TotalReads	FreeEnergy	AlignScore
ENSG00000224259	LINC01133	lincRNA	ENSG00000274712	AC005332.5	lincRNA	1	1	1	3	-13.8	13.5
Search	Search	Search	Search	Search	Search	Search	Search	Search	Search	Search	Search

D

#please cite:
#Zhou KR, Liu S, Cai L, et al. ENCORI: The Encyclopedia of RNA Interactomes.
#2.Li JH, et al.starBase v2.0: decoding miRNA-ceRNA, miRNA-ncRNA and protein-RNA interaction networks from large-scale CLIP-Seq data , Nucleic Acids Res. 2014 Jan;42:D92-7.

RBP	geneID	geneName	geneType	clusterNum	clipExpNum	clipIDnum	HepG2	K562	pancancerNum
DKC1	ENSG00000224259	LINC01133	lincRNA	2	1	2	NA	NA	10
ELAVL1	ENSG00000224259	LINC01133	lincRNA	2	2	2	NA	NA	12
FBL	ENSG00000224259	LINC01133	lincRNA	1	1	1	NA	NA	13
FUS	ENSG00000224259	LINC01133	lincRNA	6	3	6	NA	NA	10
HNRNPA1	ENSG00000224259	LINC01133	lincRNA	1	4	4	NA	NA	12
IGF2BP2	ENSG00000224259	LINC01133	lincRNA	1	1	1	NA	NA	17
NOP56	ENSG00000224259	LINC01133	lincRNA	1	1	1	NA	NA	16
PTBP1	ENSG00000224259	LINC01133	lincRNA	12	2	14	NA	NA	15
SRSF1	ENSG00000224259	LINC01133	lincRNA	1	1	1	NA	NA	15
TAF15	ENSG00000224259	LINC01133	lincRNA	4	2	4	NA	NA	11
U2AF2	ENSG00000224259	LINC01133	lincRNA	3	6	8	NA	NA	12
UPF1	ENSG00000224259	LINC01133	lincRNA	3	2	3	NA	NA	9

E

RBP	GeneID	GeneName	GeneType	ClusterNum	ClipExpNum	ClipSiteNum	HepG2(log2FC)	K562(log2FC)	Pan-Cancer
FUS	ENSG00000042286	AIFM2	protein_coding	30	4	32	-0.481	NA	17
FUS	ENSG00000156709	AIFM1	protein_coding	13	3	15	NA	NA	17
FUS	ENSG00000183773	AIFM3	protein_coding	26	3	27	NA	NA	16

RBP	GeneID	GeneName	GeneType	ClusterNum	ClipExpNum	ClipSiteNum	HepG2(log2FC)	K562(log2FC)	Pan-Cancer
FUS	ENSG00000102967	DHODH	protein_coding	4	3	4	NA	NA	26

RBP	GeneID	GeneName	GeneType	ClusterNum	ClipExpNum	ClipSiteNum	HepG2(log2FC)	K562(log2FC)	Pan-Cancer
FUS	ENSG00000167468	GPX4	protein_coding	10	3	10	NA	NA	19
Search	Search	Search	Search	Search	Search	Search	Search	Search	Search

Figure S14. The prediction of the molecules that can interact with LINC01133 .

The predicted interaction between LINC01133 and FSP1 by Lnctar (**A**) and LncRRIsearch (**B**).
(**C**) The predicted RNA binding with LINC01133.
(**D**) The predicted protein binding with LINC01133.
(**E**) The predicted binding potential between FUS and AIFM family.
