# Supplementary material for: LINC01133 can induce acquired ferroptosis resistance by enhancing the FSP1 mRNA stability through forming the LINC01133-FUS-FSP1 complex
Source: Cell Death Dis. 2023 Nov 25;14(11):767. doi: 10.1038/s41419-023-06311-z (PMC10676390; doi:10.1038/s41419-023-06311-z)
Supplement: Supplementary file 2 — Supplementary material-WB uncropped [file 41419_2023_6311_MOESM2_ESM.pdf]

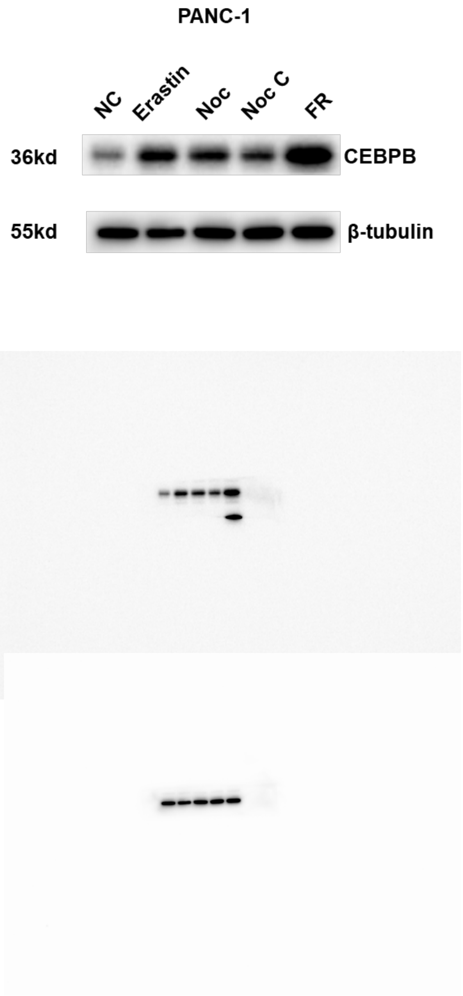

Figure 4. The regulation of CEBPB on LINC01133.
4C

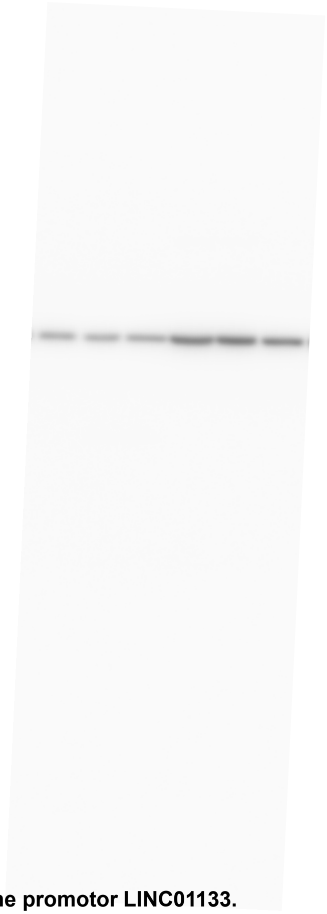

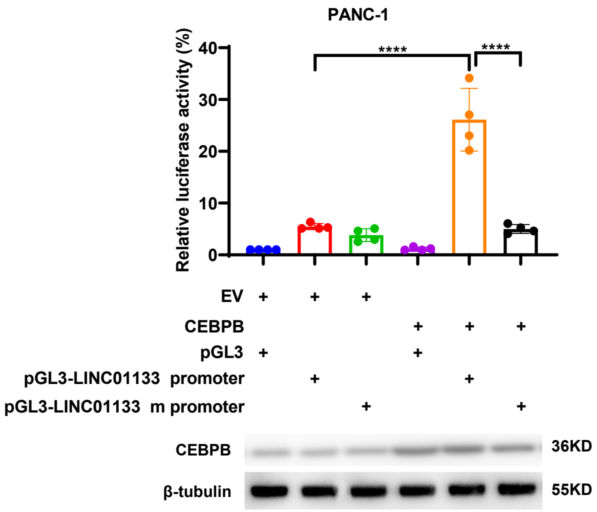

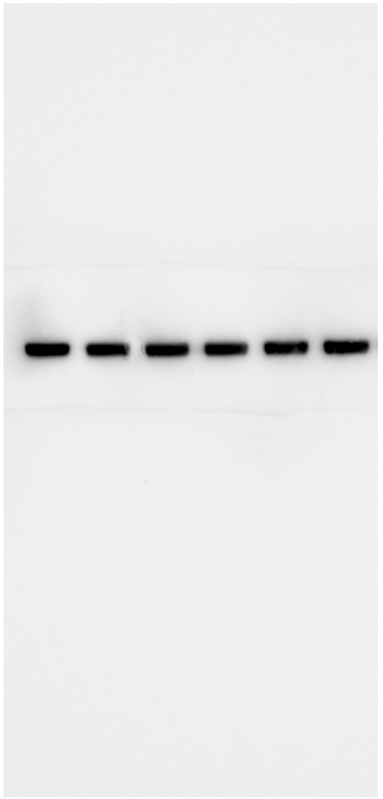

Figure 5. The binding of CEBPB to the promotor LINC01133.
5B

G-L

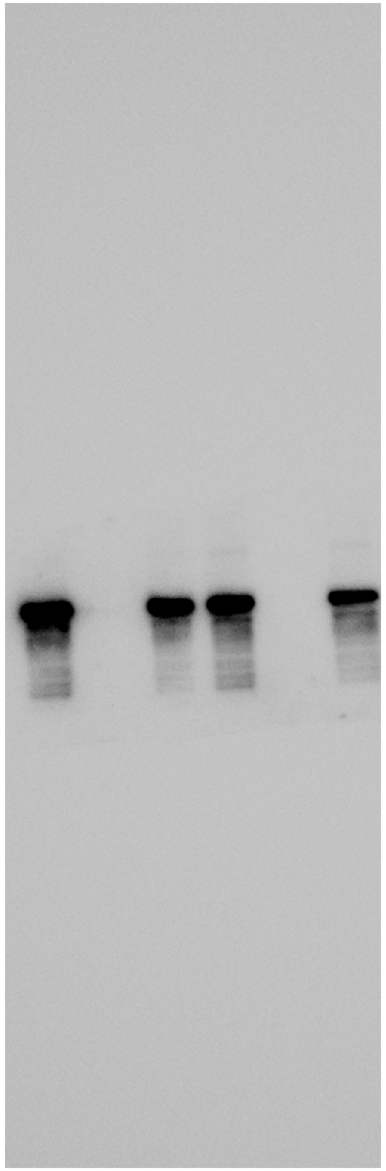

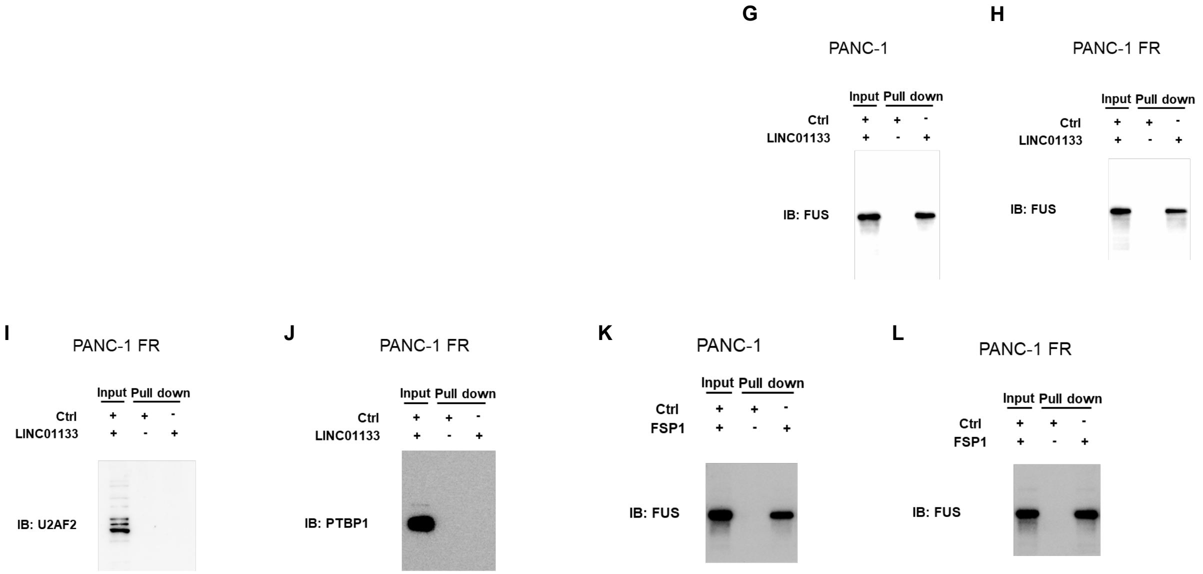

Figure 6. The validation of the LINC01133-FUS-FSP1 complex.

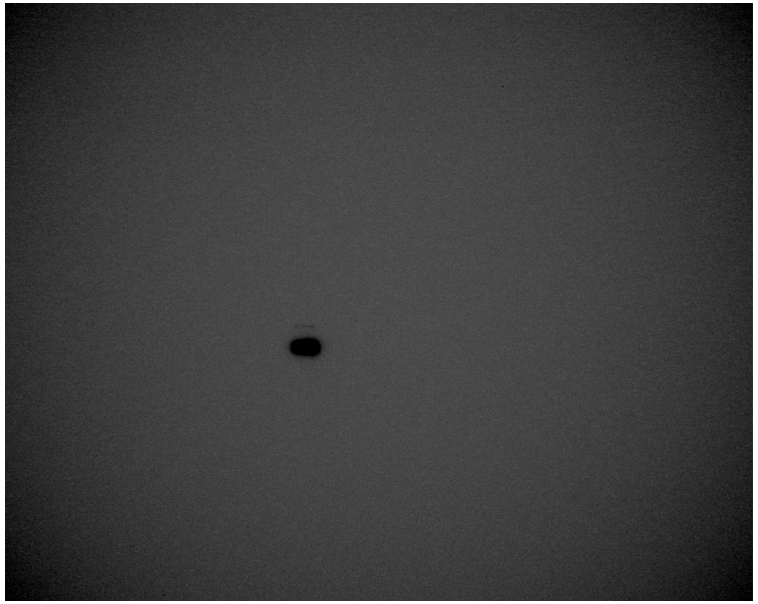

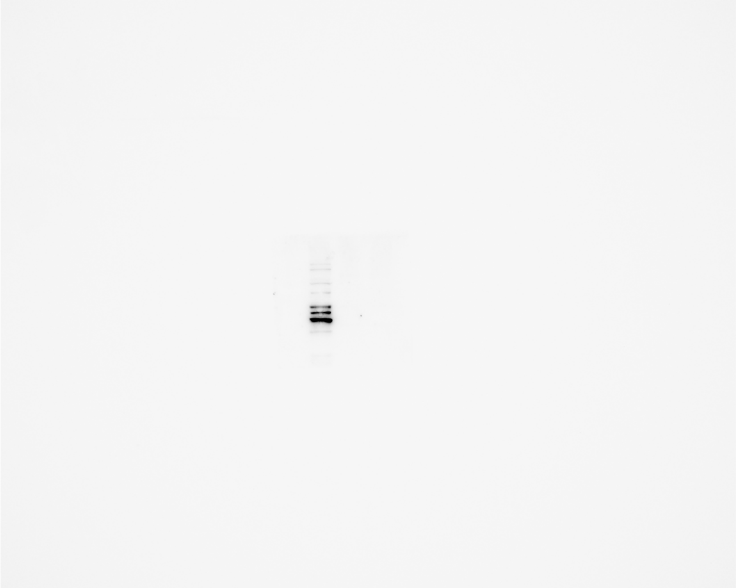

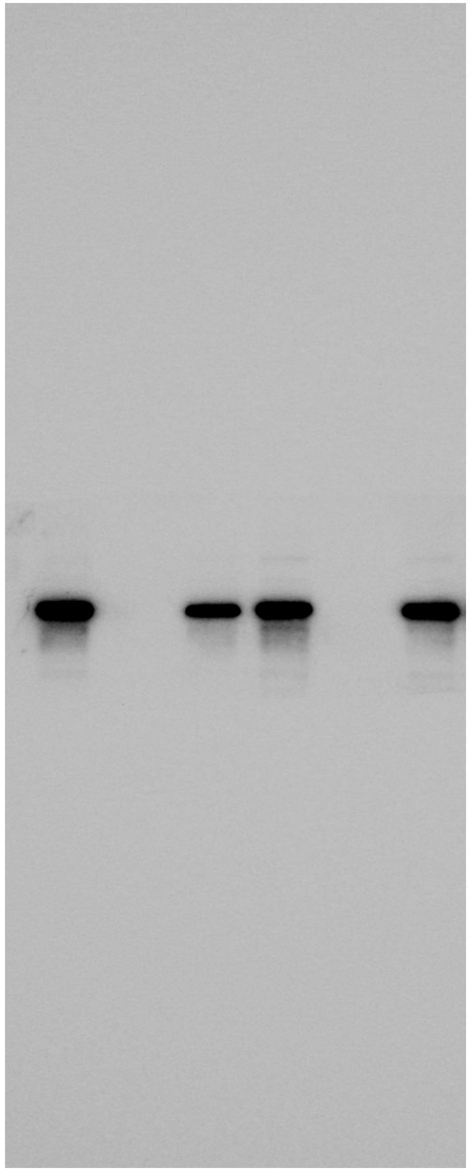

Beta-tubulin

E F

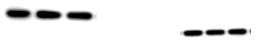

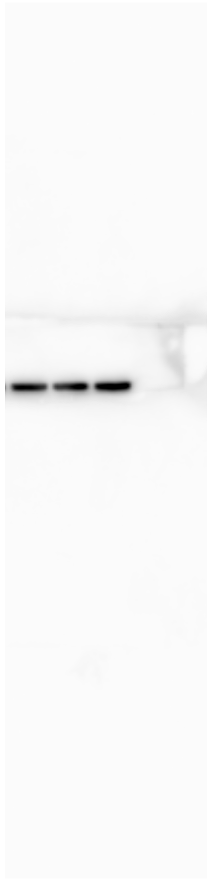

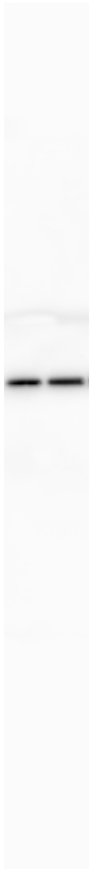

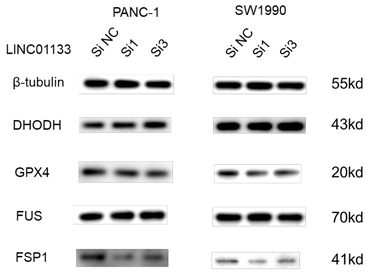

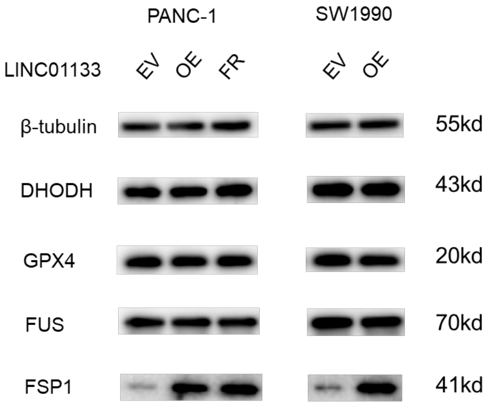

Figure 6. The validation of the LINC01133-FUS-FSP1 complex.

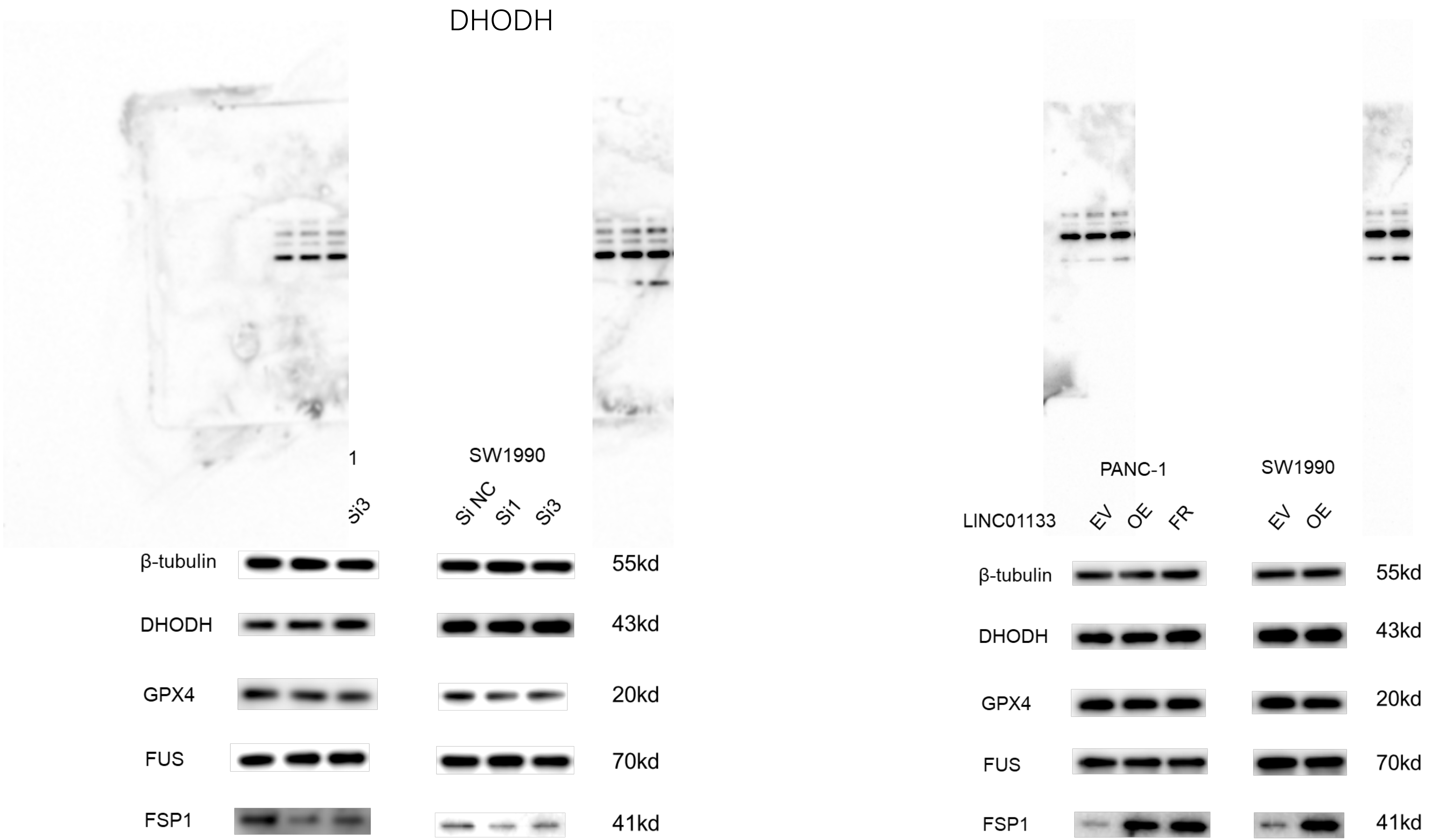
DHODH
SW1990
Si NC
Si1
Si3
β-tubulin
DHODH
GPX4
FUS
FSP1
55kd
43kd
20kd
70kd
41kd
PANC-1
SW1990
LINC01133
EV
OE
FR
EV
OE
β-tubulin
DHODH
GPX4
FUS
FSP1
55kd
43kd
20kd
70kd
41kd

GPX4

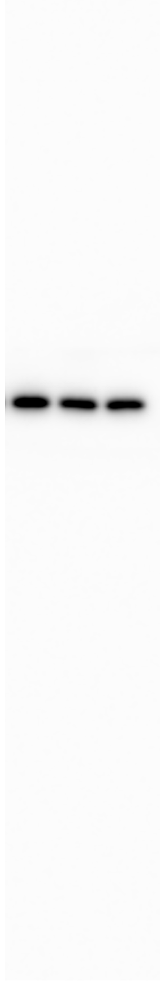

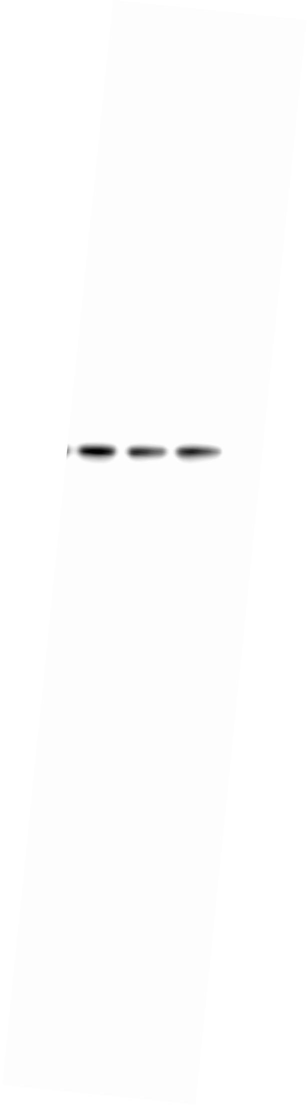

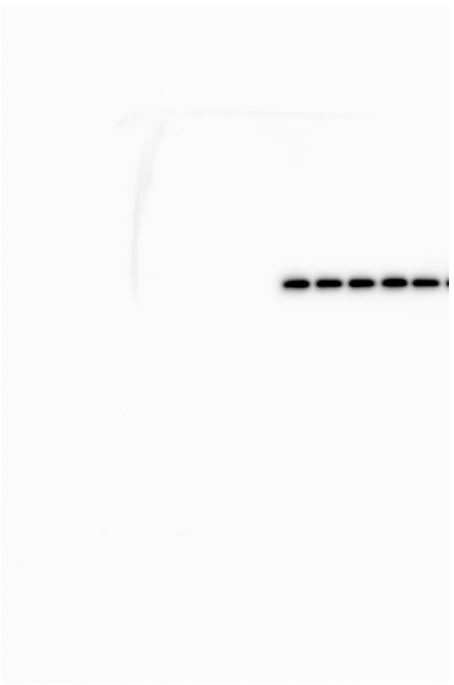

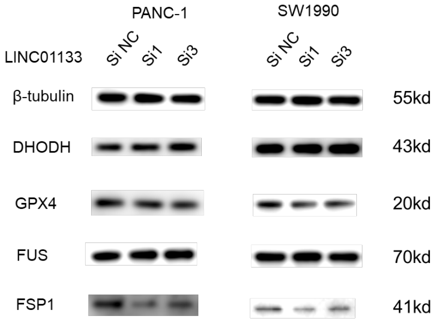
PANC-1
SW1990
LINC01133
Si NC
Si1
Si3
Si NC
Si1
Si3
β-tubulin
DHODH
GPX4
FUS
FSP1
55kd
43kd
20kd
70kd
41kd

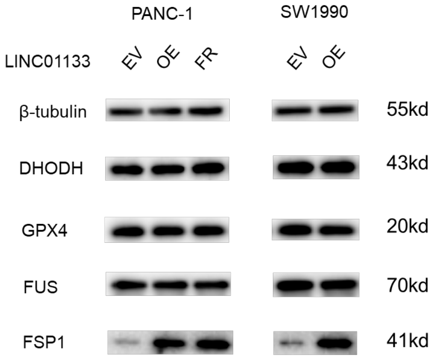
PANC-1
SW1990
LINC01133
EV
OE
FR
EV
OE
β-tubulin
DHODH
GPX4
FUS
FSP1
55kd
43kd
20kd
70kd
41kd

FUS

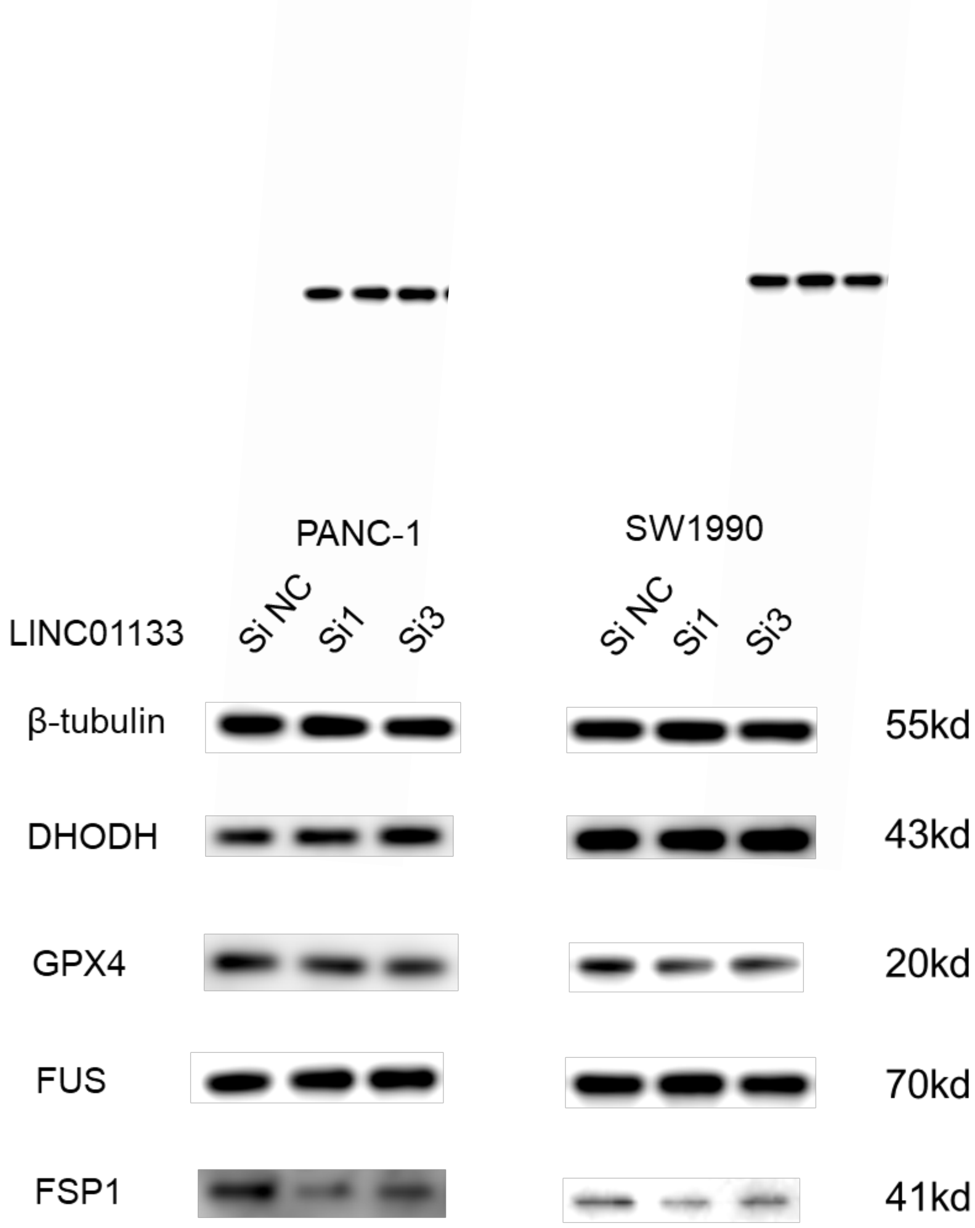
PANC-1
SW1990
LINC01133
Si NC
Si1
Si3
Si NC
Si1
Si3
β-tubulin
DHODH
GPX4
FUS
FSP1
55kd
43kd
20kd
70kd
41kd

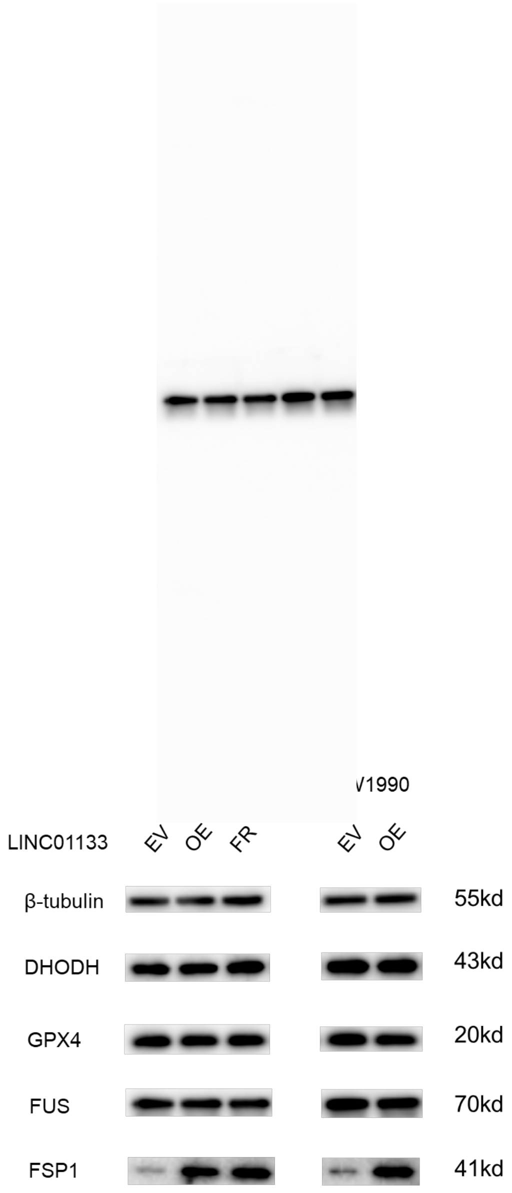
V1990
LINC01133
EV
OE
FR
EV
OE
β-tubulin
DHODH
GPX4
FUS
FSP1
55kd
43kd
20kd
70kd
41kd

FSP1

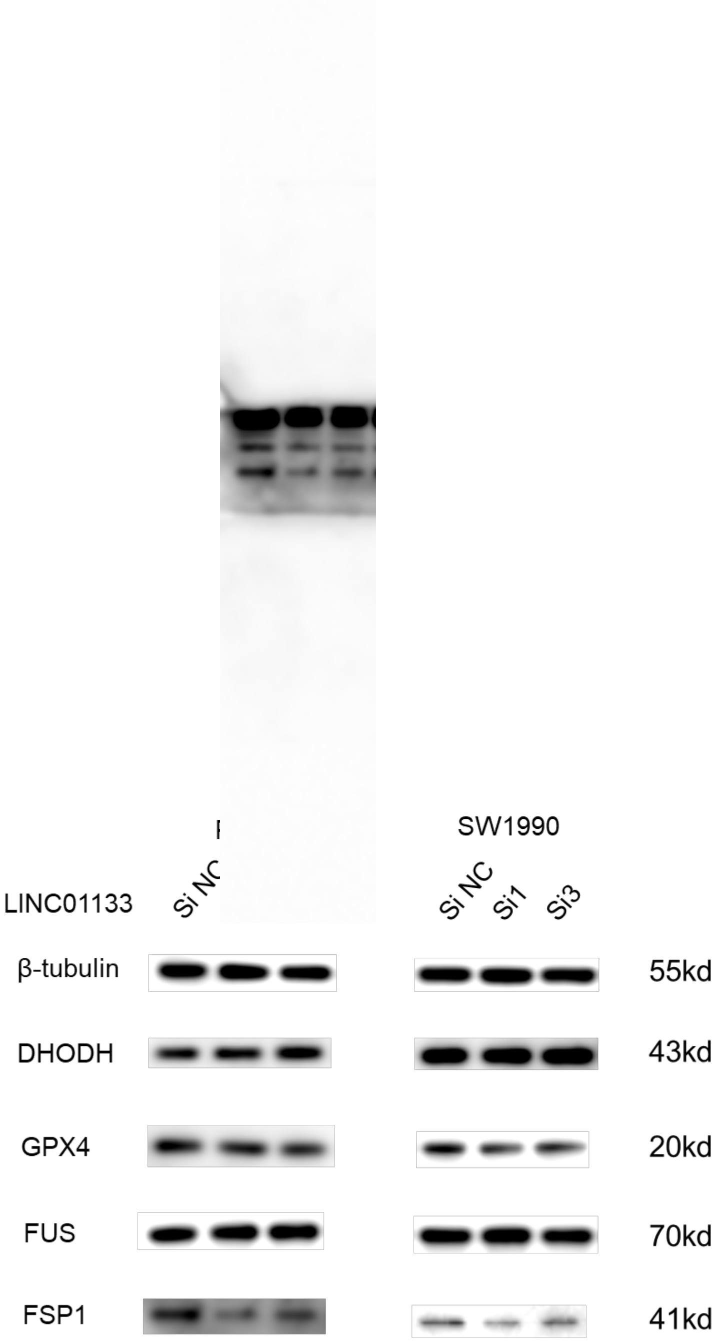
SW1990
LINC01133
Si NC
Si NC
Si1
Si3
β-tubulin
DHODH
GPX4
FUS
FSP1
55kd
43kd
20kd
70kd
41kd

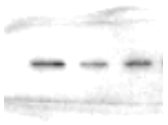

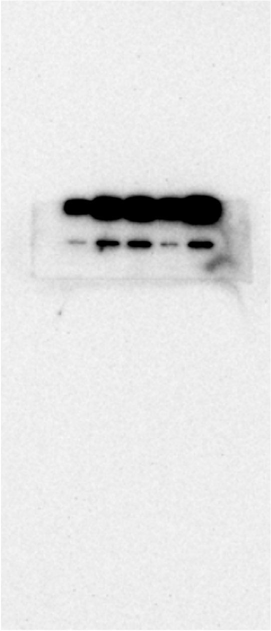

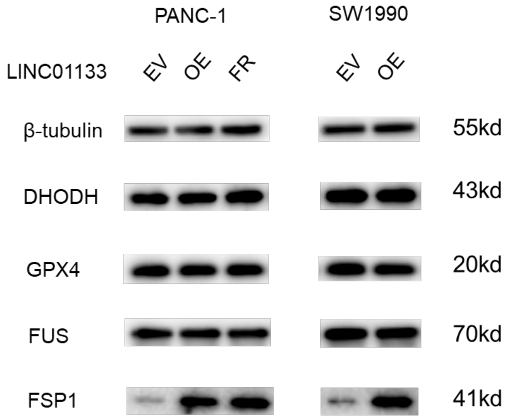
PANC-1
SW1990
LINC01133
EV
OE
FR
EV
OE
β-tubulin
DHODH
GPX4
FUS
FSP1
55kd
43kd
20kd
70kd
41kd

Figure S11. The validation of the knockdown and overexpression of CEBPB.
C F H

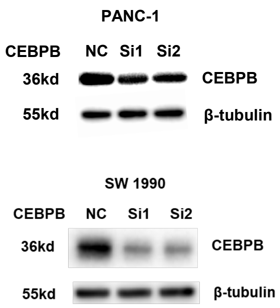

CEBPB

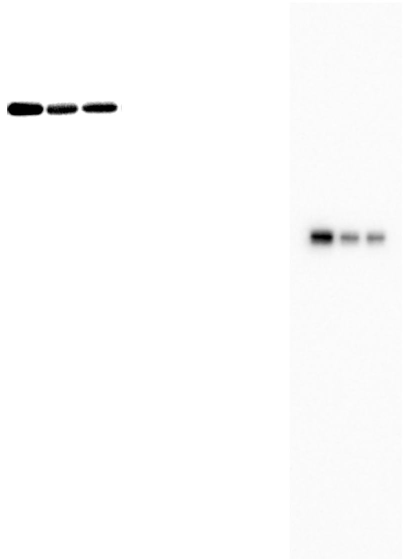

Beta tubulin

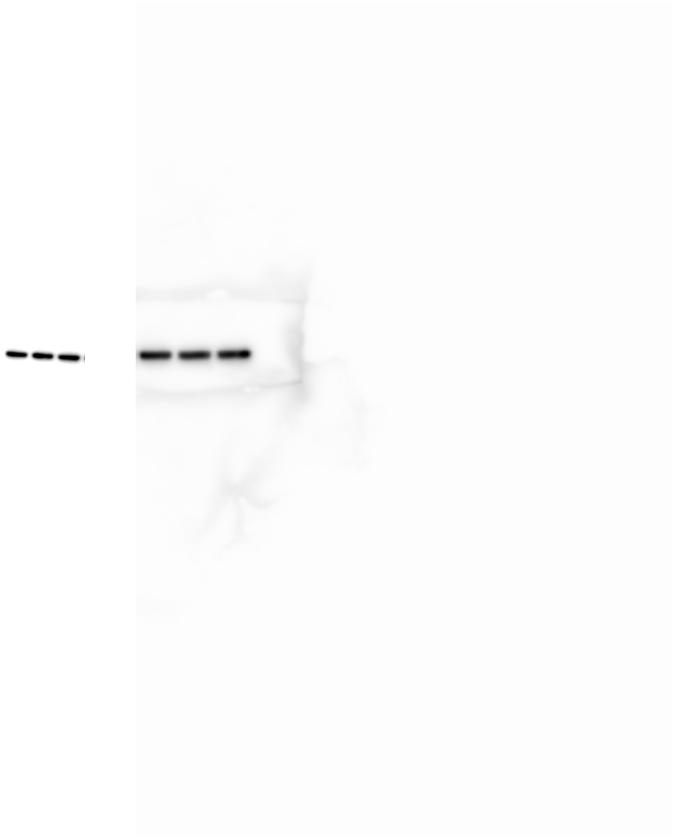

Figure S11. The validation of the knockdown and overexpression of CEBPB.
C F H

Beta tubulin

CEBPB

F

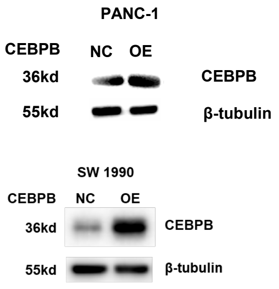

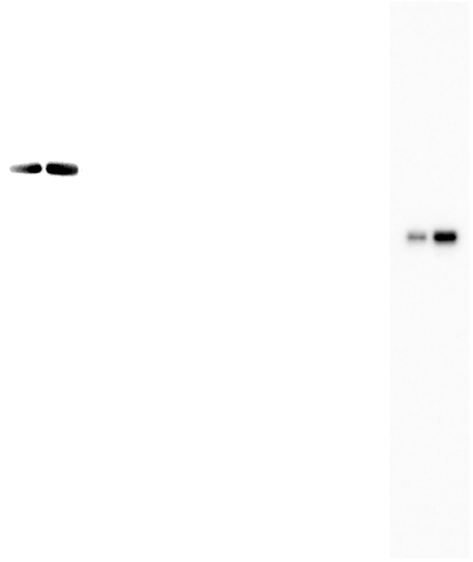

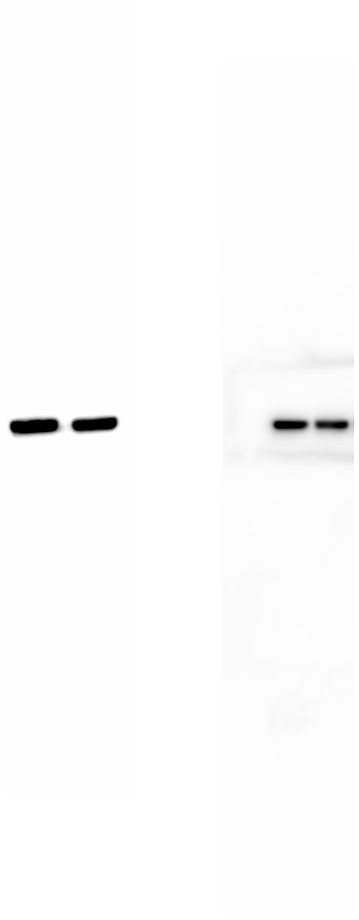

Figure S11. The validation of the knockdown and overexpression of CEBPB.
C F H

H

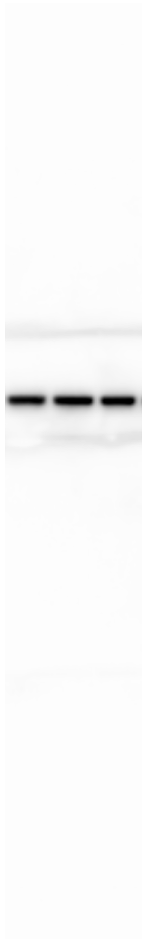

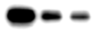

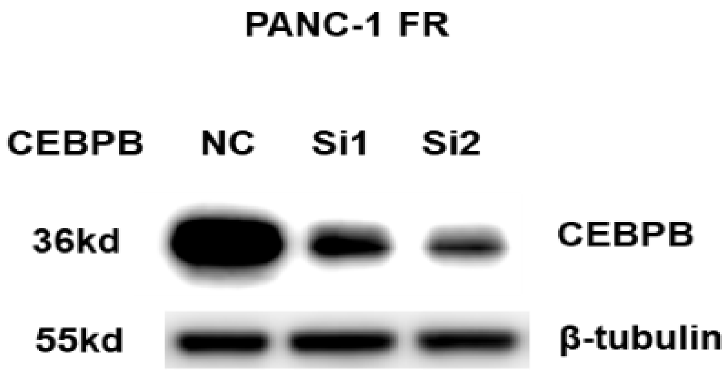

O

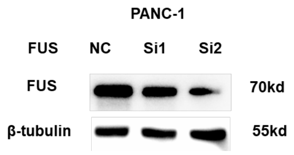

P

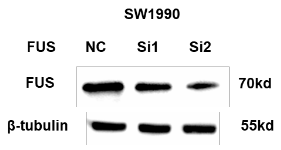

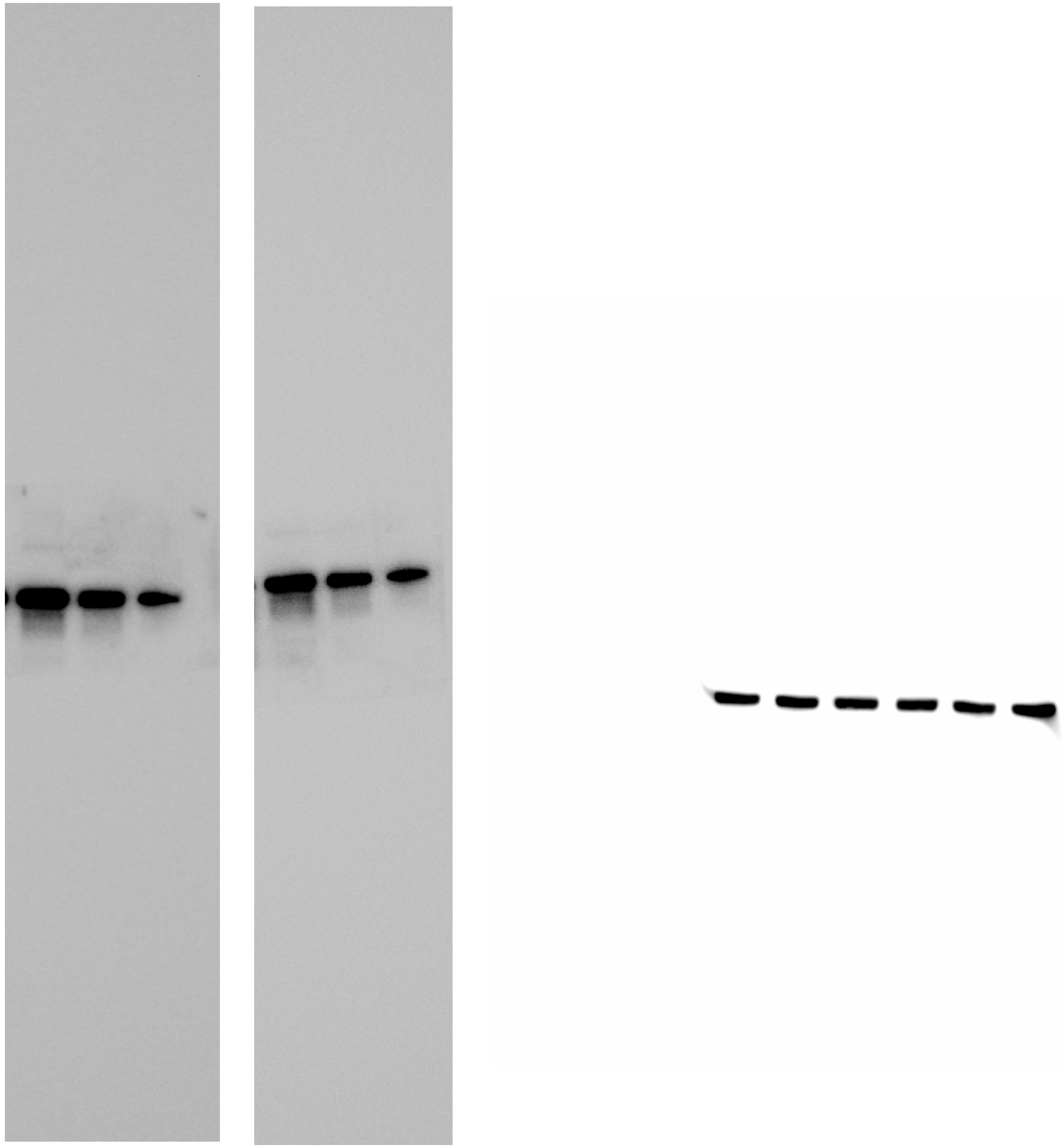

Figure S13. The vital antioxidant molecules' mRNA change by LINC01133 knockdown and the validation of FUS knockdown.
O P
